# Can REM Sleep Localize the Epileptogenic Zone? A Systematic Review and Analysis

**DOI:** 10.3389/fneur.2020.00584

**Published:** 2020-07-24

**Authors:** Graham A. McLeod, Amirhossein Ghassemi, Marcus C. Ng

**Affiliations:** ^1^Department of Clinical Neurosciences, University of Calgary, Calgary, AB, Canada; ^2^Biomedical Engineering, University of Manitoba, Winnipeg, MB, Canada; ^3^Section of Neurology, Department of Internal Medicine, University of Manitoba, Winnipeg, MB, Canada

**Keywords:** rapid eye movement sleep, epileptogenic zone, seizure onset zone, source localization, epilepsy surgery, sleep-wake cycle, epilepsy, seizures

## Abstract

Epilepsy is a common and debilitating neurological disease. When medication cannot control seizures in up to 40% of cases, surgical resection of epileptogenic tissue is a clinically and cost- effective therapy to achieve seizure freedom. To simultaneously resect minimal yet sufficient cortex, exquisite localization of the epileptogenic zone (EZ) is crucial. However, localization is not straightforward, given relative difficulty of capturing seizures, constraints of the inverse problem in source localization, and possible disparate locations of symptomatogenic vs. epileptogenic regions. Thus, attention has been paid to which state of vigilance best localizes the EZ, in the hopes that one or another sleep-wake state may hold the key to improved accuracy of localization. Studies investigating this topic have employed diverse methodologies and produced diverse results. Nonetheless, rapid eye movement sleep (REM) has emerged as a promising sleep-wake state, as epileptic phenomena captured in REM may spatially correspond more closely to the EZ. Cortical neuronal asynchrony in REM may spatially constrain epileptic phenomena to reduce propagation away from the source generator, rendering them of high localizing value. However, some recent work demonstrates best localization in sleep-wake states other than REM, and there are reports of REM providing clearly false localization. Moreover, synchronistic properties and basic mechanisms of human REM remain to be fully characterized. Amidst these uncertainties, there is an urgent need for recording and analytical techniques to improve accuracy of localization. Here we present a systematic review and quantitative analysis of pertinent literature on whether and how REM may help localize epileptogenic foci. To help streamline and accelerate future work on the intriguing anti-epileptic properties of REM, we also introduce a simple, conceptually clear set-theoretic framework to conveniently and rigorously describe the spatial properties of epileptic phenomena in the brain.

## Introduction

**Figure 1:**
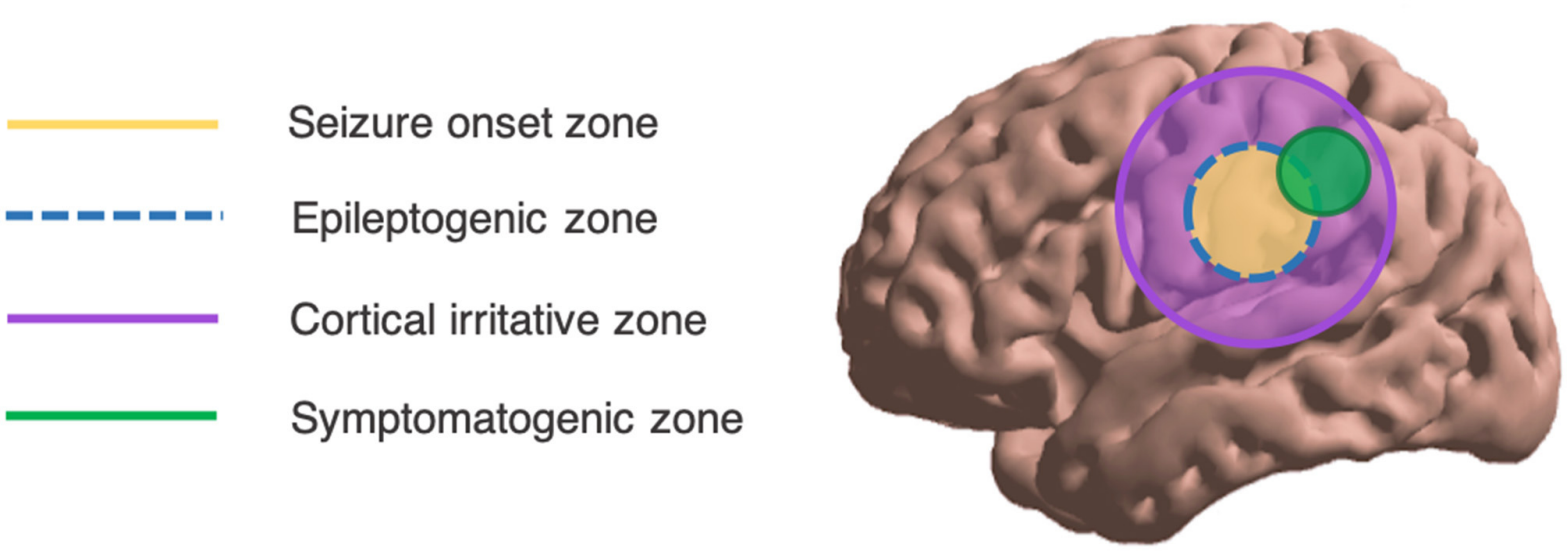
Overall schema of the spatial localization of epileptic phenomena. Ideally, the SOZ overlaps exactly with the EZ. Adapted from Lüders (9).

Epilepsy is one of the most common neurological diseases, with a new case diagnosed every 3.5 min on average in the USA alone (1). Partly due to substantially increased risk of premature death (2), epilepsy reportedly accounts for a worldwide loss of 13 million disability-adjusted life years (3). For up to 40% of persons with epilepsy, medication alone fails to control seizures (4, 5). In such cases, surgical resection of epileptogenic cortex is an effective and economical therapy to achieve seizure freedom (6–8). To guide resection, exquisite localization of the epileptogenic zone (EZ) is required, where the EZ is defined as the minimum region of brain tissue whose resection is necessary and sufficient to bring seizure freedom [Figure 1; (10)]. At the same time, localization must be highly specific to maximally preserve eloquent cortical networks.

**Figure 2:**
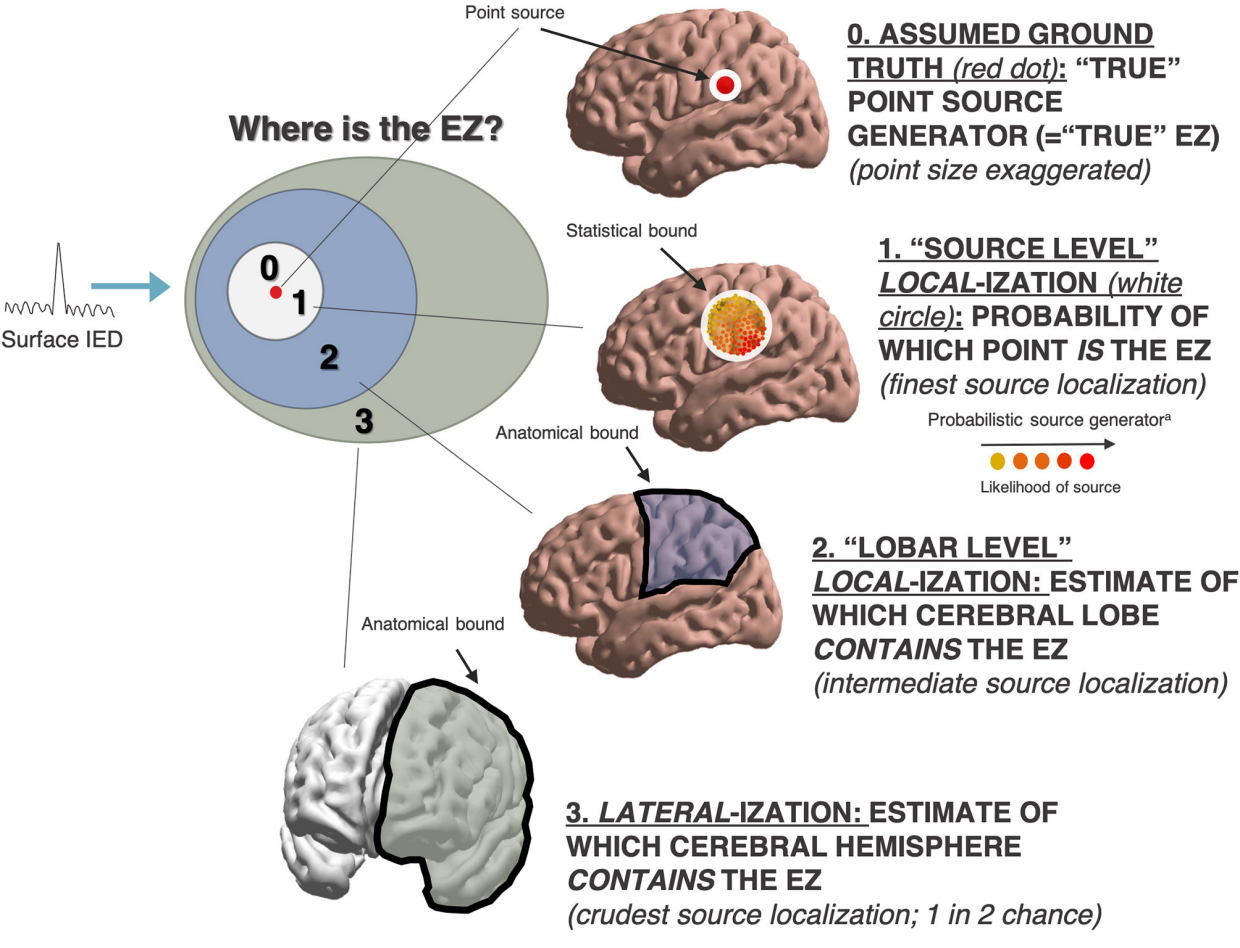
Heterogeneous measures in interictal source localization. ^a^Probabilistic source location not necessarily confined to any one anatomic lobe (i.e. as a finer and more granular form of source localization).

From standard scalp electroencephalography (EEG), spatial localization of intracranial seizure generators is challenging. Ictal events may be missed such that interictal phenomena like interictal epileptiform discharges (IEDs) and high-frequency oscillations (HFOs) are relied upon. However, these tend to occupy a greater spatial expanse than the EZ or the seizure onset zone (SOZ; see Figure 1). Further, scalp EEG best detects cortical foci that are near to the surface, with a preference for temporal lobe foci (11); deeper foci may be missed. From surface potentials, there is the possibility of intracranial source reconstruction via sophisticated modeling techniques including minimum norm (MN) imaging and low-resolution brain electromagnetic tomography analysis (LORETA). However, these techniques require solving the inverse problem—which states that a given scalp surface potential could have arisen from any of an infinite set of potential source generators (12)—and are not in widespread clinical use (Figure 2). Even clinical semiology of ictal events is of varying reliability, as the symptomatogenic cortex may imperfectly overlap with the EZ or SOZ (Figure 1).

Despite challenges of triangulating intracranial foci from scalp recording, occasionally a reliable localization can be obtained from scalp EEG phenomena [in conjunction with other clinical data such as brain magnetic resonance imaging (MRI)], and such patients proceed directly to surgery. However, many surgical candidates must undergo invasive intracranial EEG recording (iEEG). In such cases, to guide implantation of invasive electrodes, accurate preliminary localization by surface phenomena is still required. Moreover, localizing with iEEG is also non-trivial, as it is impossible to obtain perfect coverage of all cortical tissue. Thus, there is a desire for recording and analytical techniques that can improve the localization yield from interictal epileptic phenomena.

The notion that different sleep-wake states may differentially localize epileptogenic foci dates to at least the 1940s (13). Sleep and epilepsy are long known to be interrelated (14), with recent work illuminating many details of the relationship (15–17). Regarding the localization of epileptogenic foci, rapid eye movement sleep (REM) is of particular interest. In REM, IEDs are known to be of shorter duration, duller contour, and lower amplitude (18–21). Moreover, IEDs in REM are rare (22), but reports dating back to the 1970s−1980s suggest they may be of high localizing value (23–25). Certain recent work continues to indicate a localizing role for REM (26–29); however, other work reports alternative sleep-wake states to be of higher localizing value (30, 31). Further, REM has been noted to occasionally provide “false localization” to a non-veridical SOZ (21, 32). However, there is an intriguing mechanistic argument to be made on grounds of cortical asynchrony that REM may suppress propagation of epileptic phenomena, constraining them spatially to regions proximate to the true source generator (33). It is further thought that this mechanism may account for recently reported abilities of REM to shrink overall field size of IEDs (26) and pathological HFOs (34).

To consider the important questions of whether and how REM may help localize epileptogenic foci, we systematically reviewed the literature on epileptic phenomena in REM. Where possible, we conducted quantitative analyses to synthesize insights from heterogeneous reports.

## Methods

### Literature Review

We sought original research investigating spatial attributes or localizing value of epileptic phenomena in REM in humans. To this end, we searched broadly in PUBMED, Scopus, and EMBASE in March 2020 (see Supplementary Information for detailed search strategy including search terms). All abstracts were screened (GAM) for pertinence according to the following inclusion criteria:

1. Original research in human subjects.
2. Epileptic phenomena recorded in REM.
3. Spatial attributes of the epileptic phenomena in REM were analyzed.

To capture literature meeting above criteria, studies were subject to full-text analysis if they appeared to consider the localization of epileptogenic foci vis-à-vis sleep-wake states. Full texts were then independently analyzed by two authors (GAM, MCN).

### Quantitative Analyses

To aid interpretation of heterogeneous literature and further characterize the effects of REM on spatial attributes of epileptic phenomena, we sought to perform quantitative analyses of select pertinent records meeting additional, rigorous criteria. Additional criteria were as follows:

4. Per-patient data available for majority of subjects.
5. *N* >3 subjects (essentially, not a case report).

We aimed to conduct four distinct quantitative analyses: (1) localization value of interictal epileptic phenomena during REM as arbitrated by clinic SOZ or (2) by gold standard of post-resective surgery seizure freedom; and (3) relative spatial extent and (4) spatial novelty of REM IED field. When we considered seizure freedom, we adopted the metric used by the study in question; for example, complete seizure freedom as per the International League Against Epilepsy (ILAE) Surgery Outcome Classification (35), or freedom from disabling seizures as per Engel Class I (36). For convenience, we will simply refer to all post-operative seizure freedom metrics as “Engel Class I.” For all analyses, we weighted per study rather than per patient, such that 1/5 (=20%) patients in one study was weighted equivalently to 10/50 (=20%) patients in another study. Where possible, we also performed subgroup comparisons of surface vs. intracranial recording techniques, and lateralization vs. localization metrics.

## Results

### Summative Literature Review

**Figure 3:**
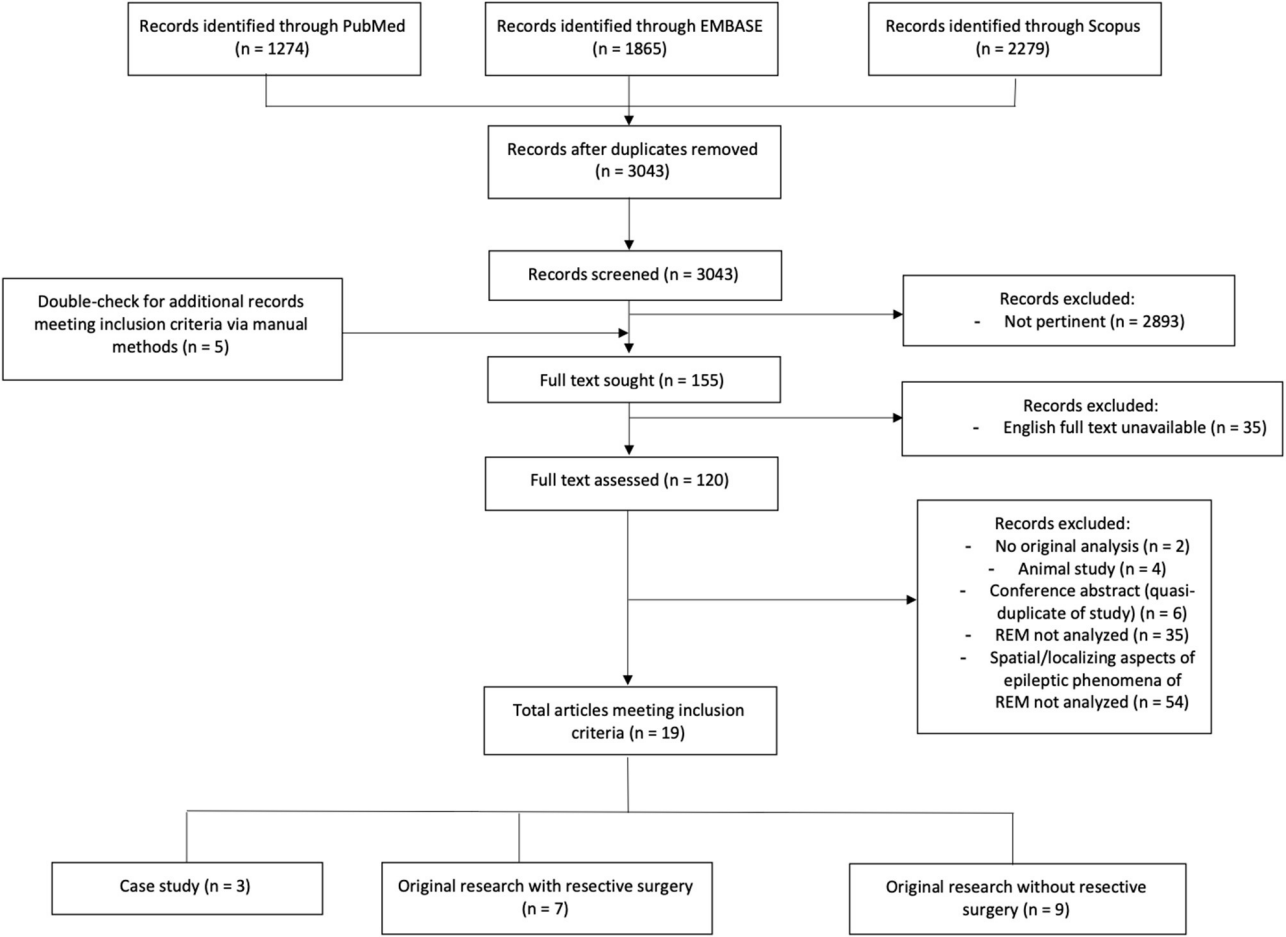
Literature extent of research in spatial attributes or localization value of epileptic phenomena in REM. Flowchart showing results of our literature search according to PRISMA criteria. Detailed search strategy is documented in Supplementary Information. PRISMA: preferred reporting items for systematic reviews and meta-analyses.

After searching broadly in PUBMED, Scopus, and EMBASE, we obtained 3,043 unique records (see PRISMA diagram in Figure 3). From screening of abstracts, 120 advanced to full-text analysis. From 120 full texts, articles were discarded because they were animal studies, review papers, or a duplicate of a record already obtained; or because they did not consider REM sleep, or did not consider a specific localizing or spatial attribute of REM (see Figure 3 for exact counts and exclusions). Nineteen original research studies were ultimately deemed pertinent (Table 1).

**Table 1:** Overview of studies investigating spatial attributes of epileptic phenomena in REM sleep.

**References**	**Subjects**	**Epileptic phenomena**	**Spatial assessment**	**Localization arbitrated by**	**Finding**
(26)	Medically-refractory focal epilepsy (*n* = 6)	Scalp IEDs or slowing	Number of involved EEG channels enumerated. Source localization performed by MN imaging and cMEM, spatial extent quantified	Clinical SOZ from EMU	In group-level analyses, spatial extent of IED scalp field and source generation was reduced in REM compared to NREM. IEDs in REM co-localized with clinically-determined SOZ in 4/6 of patients (NREM co-localized in 3/6 patients).
(31)	Medically-refractory focal epilepsy and resective surgery (*n* = 30)	Stereo-EEG oscillatory events (including HFOs), univariate spectral analysis, bivariate connectivity measures, and IEDs	Stereo-EEG contacts inside resected zone, or within 5 cm thereof	Zone of resection; post-surgical outcome	NREM2 and 3 contained features that localize the epileptogenic zone with superior accuracy compared to other states including REM.
(34)	Medically-refractory focal epilepsy (*n* = 15)	Intracranial stereotactic EEG pathologic ripples (HFOs of 180–250 Hz, restricted to channels in the IZ and/or SOZ)	Involved channels enumerated	IZ and SOZ as per intracranial recording	In REM, pathological ripples occupied a smaller spatial field than in NREM2 and 3 (awake and NREM1 not assessed).
(37)	Focal or generalized epilepsy (*n* = 69)	Scalp IEDs	Location of IEDs	N/a	REM clarified localization in 7 patients, shrinking IED field in 6.
(38)	Medically refractory MTLE (*n* = 56)	Scalp IEDs	Lateralization of IEDs	Hemisphere with temporal lobe lesion on MRI	In 4/5 patients with bilateral NREM IEDs, REM IEDs lateralized concordant to MRI lesion.
(28)	Lateralized ictal onset but generalized IEDs and non-lesional MRIs (*n* = 20); resective surgery (*n* = 17)	Scalp IEDs	Lateralization to epileptic hemisphere	Hemisphere of resection; post-surgical outcome.	Scalp IEDs in REM lateralized to epileptic hemisphere in 15/20 patients, vs. NREM IEDs in 3/20 and awake IEDs in 10/20; 13/17 patients Engel I.
(27)	Medically-refractory focal epilepsy and resective surgery (*n* = 12)	Individual intracranial electrodes classified as displaying HFOs pre-dominantly in REM, NREM, or neither	Electrodes inside or outside zone of resection	Zone of resection; post-surgical outcome	In patients with post-operative seizure freedom, REM-predominant-HFO electrodes were statistically associated with zone of resection, but NREM-predominant-HFO electrodes were not.
(21)	Medically-refractory focal epilepsy (*n* = 70)	Scalp IEDs	Location of IEDs	SOZ as per pre-surgical scalp-video EEG evaluation	NREM IEDs had highest localizing value. REM IEDs provided additional localizing data in 12 patients, concordant with other data in 7, discordant in 5.
(39)	Medically-refractory TLE (*n* = 13); some MTLE (*n* = 9), others neocortical (*n* = 4)	Intracranial depth and subdural IEDs	Lateralization to epileptic hemisphere	SOZ as per pre-surgical scalp-video EEG evaluation	10/11 patients had 75% of intracranial IEDs lateralized to epileptic hemisphere in REM and wakefulness, vs. 8/11 in NREM1/2 and 9/11 in NREM3.
(29)	Medically-refractory epilepsy secondary to TSC (*n* = 23); ictal EEG lateralized to one hemisphere (*n* = 15); resective surgery (*n* = 13)	Scalp IEDs	Lateralization to epileptic hemisphere	Ictal semiology; ictal EEG; lateralization of largest tuber; hemisphere of resection; post-surgical outcome	REM outperformed NREM and wakefulness in lateralizing to the side of ictal EEG, seizure semiology, largest tuber, and resected hemisphere.
(40)	Focal epilepsy (*n* = 6)	Scalp IEDs	Anatomic lobes in which IEDs were observed	n/a	In 3 patients, IEDs in REM occupied novel spatial fields.
(41)	Medically refractory MTLE (*n* = 1)	Scalp and depth IEDs	Location of IEDs	Left temporal region was resected with post-operative seizure freedom	All scalp IEDs in REM lateralized to left temporal region. IEDs in NREM lateralized predominantly to R temporal region; ictal EEG (scalp and depth) showed independent bilateral ictal onsets.
(18)	Medically-refractory MTLE (*n* = 21)	Scalp IEDs	Bilateral vs. unilateral	n/a	Only 1 subject had bilateral IEDs in REM, whereas 10 subjects did in NREM.
(42)	Generalized or focal epilepsy (*n* = 188)	Scalp seizures	EEG secondary generalization	n/a	Of 5 focal-onset seizures in REM, 0 generalized (vs. 67/189 in NREM and 77/428 in wakefulness).
(32)	Landau-Kleffner Syndrome (*n* = 1)	Scalp IEDs	Location of IEDs	n/a	IEDs in REM spread bilaterally (less localizing).
(19)	Medically-refractory TLE (*n* = 37) and resective surgery (*n* = 32)	Scalp IEDs	Lateralization to epileptic hemisphere	Final localizing diagnosis as per pre-surgical evaluation	All REM IEDs were unilaterally concordant with epileptic hemisphere.
(24)	Generalized or focal epilepsy (*n* = 43) and resective surgery (*n* = 12)	Scalp IEDs	Association with zone of resection	Zone of resection, post-surgical outcome	REM's localization concordant with zone of resection in 7/8 Engel I patients.
(23)	Medically-refractory TLE (*n* = 10) and resective surgery (*n* = 9)	Depth IEDs	Lateralization to epileptic hemisphere	Hemisphere of resection, post-surgical outcome	REM lateralized to hemisphere of resection in 7/9 patients, 4 of whom were Engel I.
(25)	Medically refractory MTLE (*n* = 8) and temporal lobe seizures (*n* = 1)	Scalp IEDs (*n* = 9) and depth IEDs (*n* = 1)	Location of scalp IEDs	n/a	In 1 patient, scalp IEDs in REM originated from a unique focus.

*cMEM, coherent minimum entropy on the mean; EEG, electroencephalography; EZ, epileptogenic zone; HFO, high-frequency oscillations; IED, interictal epileptiform discharge; IZ, irritative zone; MN, minimum norm; MTLE, mesial temporal lobe epilepsy; NREM, non-rapid eye movement sleep; REM, rapid eye movement sleep; SOZ, seizure onset zone; TLE, temporal lobe epilepsy*.

**Figure 4:**
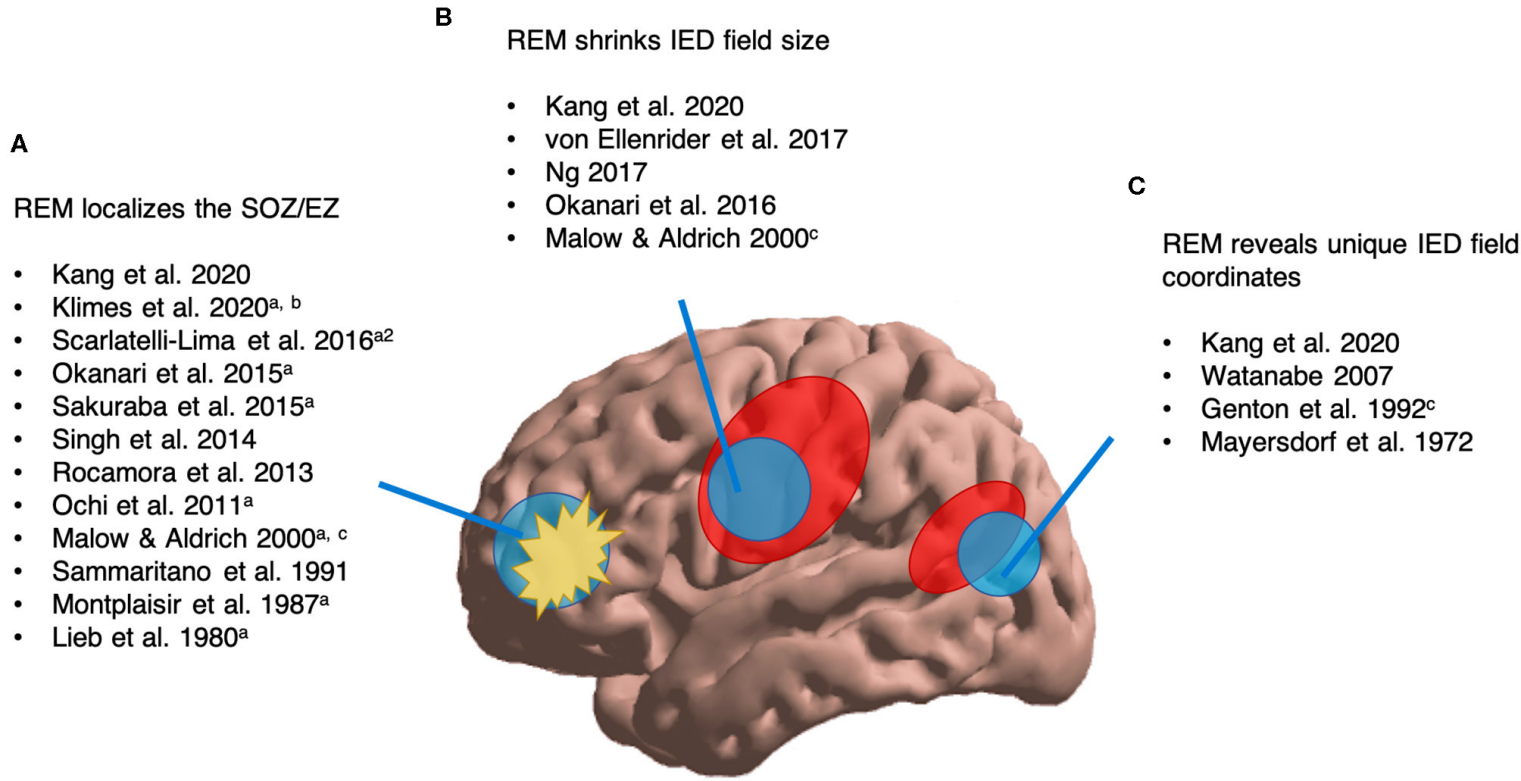
Overview of available literature assessing REM's interictal localizing value of epileptic phenomena. ^a^Localization validated by post-surgical seizure freedom. ^a2^Surgical outcomes reported for 4/56 patients. ^b^NREM/awake outperformed REM. ^c^Case study (*n* = 1–2). **(A)** We found 12 studies assessing REM's ability to localize the SOZ/EZ. **(B)** We found five studies reporting that REM can shrink IED field size. **(C)** We found four studies suggesting that REM can reveal unique IED field coordinates.

**Figure 5:**
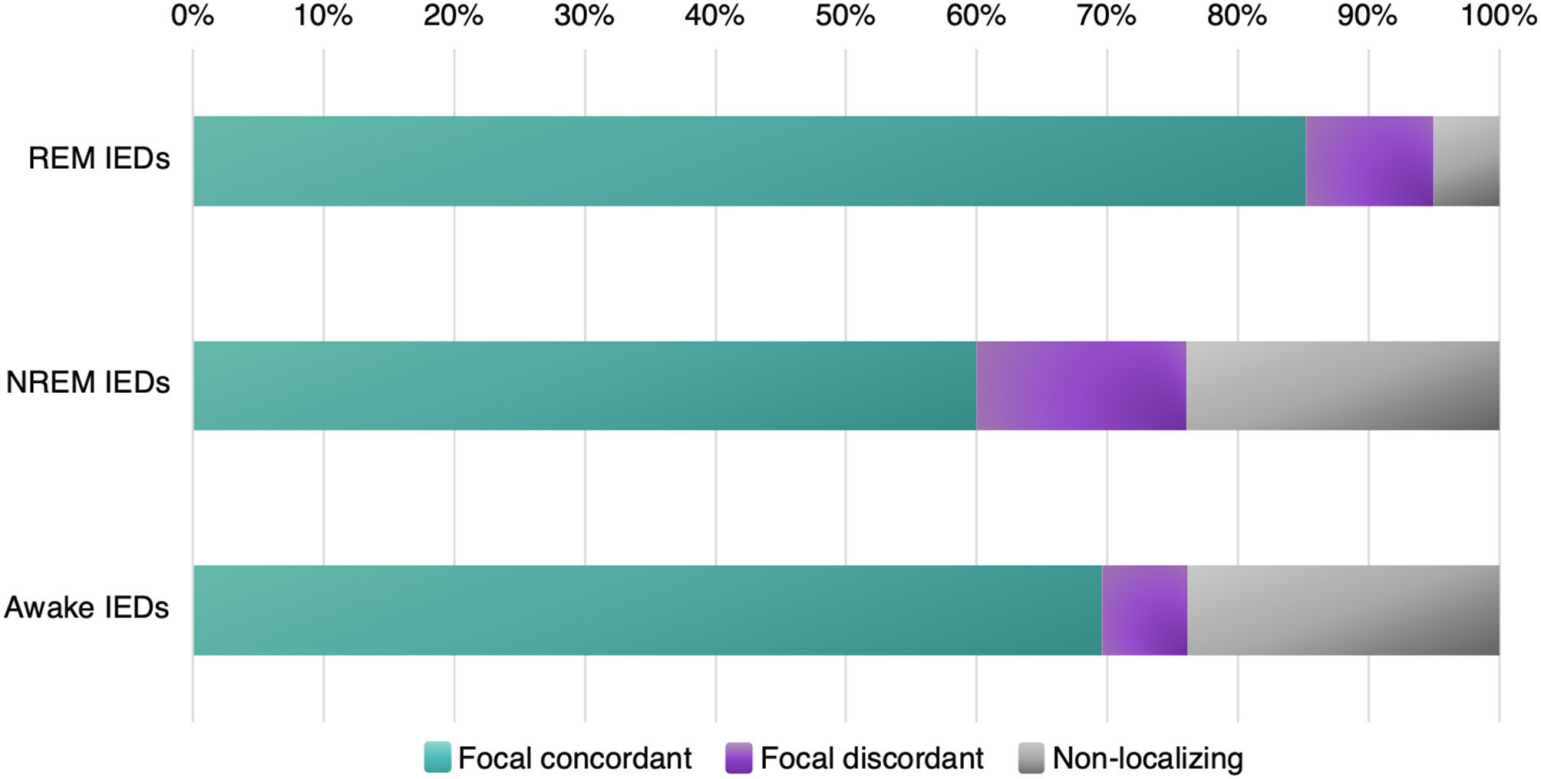
REM's ability to localize the clinical SOZ.

**Figure 6:**
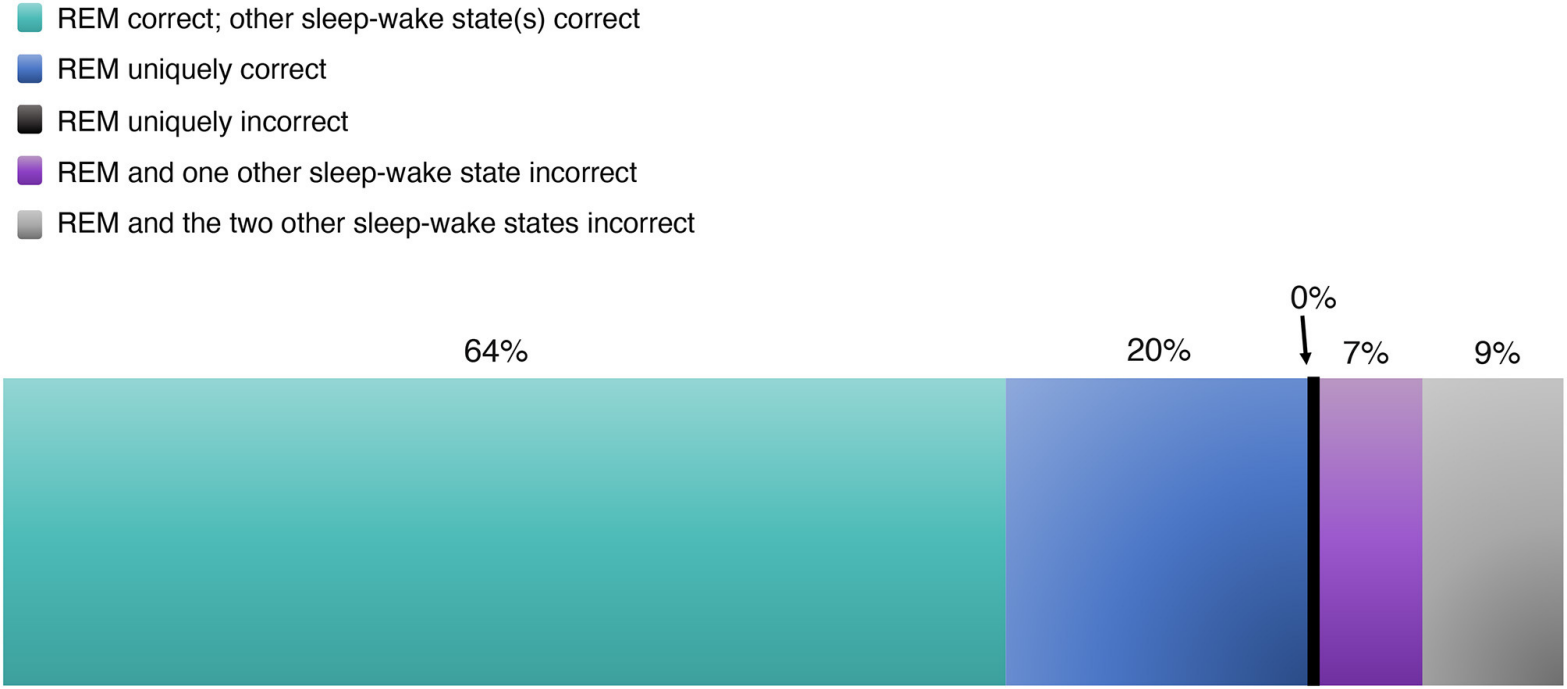
REM's ability to localize the EZ as validated by post-operative seizure freedom.

**Figure 7:**
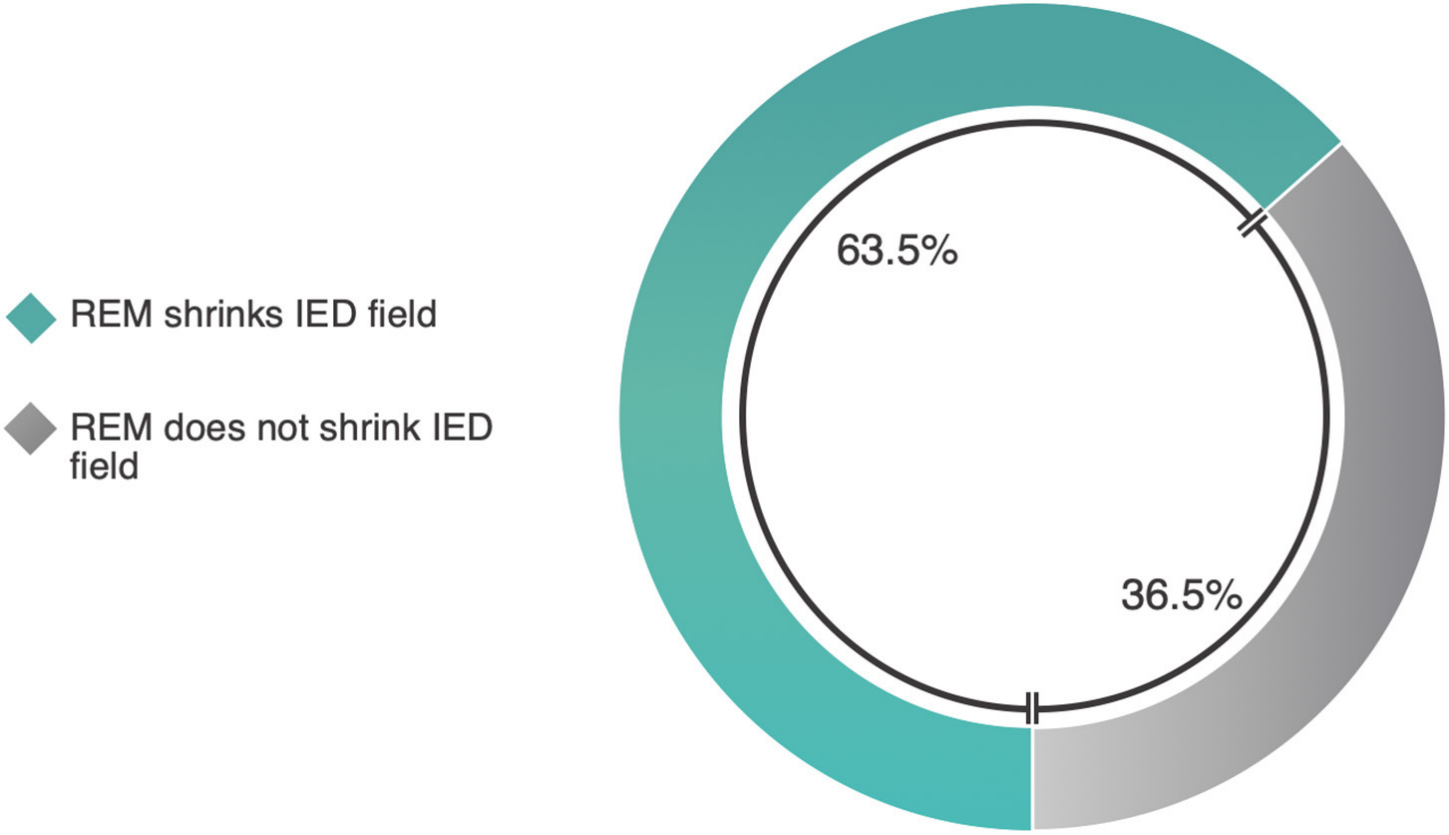
REM's ability to shrink the IED field.

**Figure 8:**
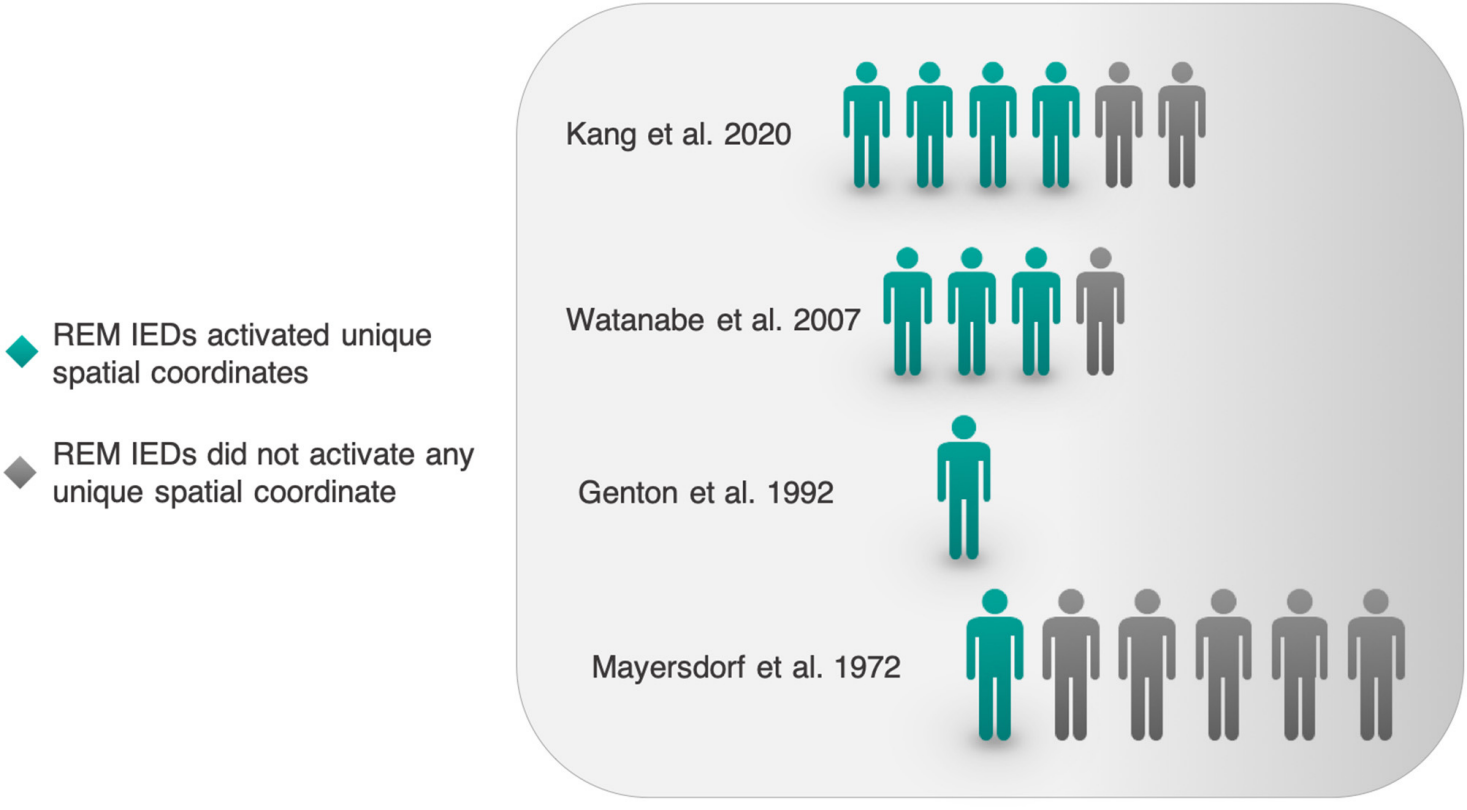
REM can activate unique IED field spatial coordinates.

Of 19 pertinent studies, one focused on ictal phenomena (42); the remainder focused on interictal epileptic phenomena. In reviewing 18 interictal studies, three main themes emerged (Figure 4): (1) using REM to localize the EZ or SOZ, (2) REM shrinking the IED field, (3) REM IEDs occupying new spatial coordinates. As follows, we conducted quantitative analyses of spatial and localizing attributes of interictal epileptic phenomena in REM (sections Interictal Localization by REM Compared to Clinical SOZ, Interictal localization by REM as Arbitrated by Post-resective Surgery Seizure Freedom, Effect on Post-operative Seizure Freedom of Including or Excluding Interictal Sleep-Wake State Localization in a Resection, REM's Effects on IED Field Size, and REM's Effects on Spatial Novelty of the IED Field; Figures 5–8). 14/19 studies were included in one or more quantitative analyses. Six studies contained information pertinent to REM's localization value but are not included in our quantitative analyses.

### Interictal Localization by REM Compared to Clinical SOZ

#### Literature Review

For quantitative analysis, we first sought studies comparing the interictal localization of REM with the clinically-localized SOZ. Amongst articles listed in Table 1, we identified five studies (collective 91 subjects) meeting criteria for this analysis. There was some heterogeneity amongst studies included, so we will briefly describe them.

Kang et al. (26) compared IEDs and clinical SOZs by the metric of lobar correspondence. Source reconstruction was performed on surface IEDs, yielding a probability density of possible cortical generators (see Figure 2). Locations of cortical generators were then compared with lobe of ictal onset on surface EEG. Uniquely, Kang et al. reported localization proper; other studies reported lateralization.

Rocamora et al. used depth and subdural EEG to compare lateralization of intracranial IEDs with reference to a pre-surgical intracranial localization of the SOZ. These authors subdivided non-REM (NREM) into NREM1, NREM2, and NREM3. To avoid a potential bias in favor of REM, for our calculations we considered the localization of NREM3 specifically, as it marginally outperformed NREM1 and NREM2.

Whereas Kang et al. localized in a reconstructed source space, and Rocamora et al. recorded intracranially, the remaining three studies analyzed distribution of the surface IED field based on EEG-MRI co-localization, concordance with ictal EEG onset, etc. Sammaritano et al. compared lateralization of REM's scalp IED field against a “final localizing diagnosis” established in part by iEEG (19). Okanari et al. validated the lateralization of REM with post-operative outcomes (28), but post-operative outcomes were not reported per patient, so we simply considered whether REM lateralized to the side in which the pre-surgical subdural grid was implanted. 13/17 surgical patients were rendered seizure-free, indicating overall accuracy of localization. Ochi et al. performed resective surgery and reported post-operative outcomes per patient (29), but onset of ictal EEG was also reported. In our Quantitative Analysis, we include Ochi et al. solely to consider the correspondence of REM's localization with the clinical SOZ, agnostic to post-operative outcomes.

In our calculations (Table 2) we considered IEDs in REM, NREM, and wakefulness; and then categorized them as focal concordant (localizing to the clinical SOZ), focal discordant (localizing somewhere other than the clinical SOZ), or non-localizing (generalized, diffuse, bilateral, etc.). For each sleep-wake state, we considered the proportion of subjects whose IEDs fit into each category (focal concordant; focal discordant; non-localizing) vs. the total number of subjects displaying IEDs in that state (rather than against the total number of subjects in the study).

**Table 2:** Interictal localization of REM arbitrated by clinical SOZ.

**IEDs**	**Focal concordant** ***n*** (%)^*^			**Focal discordant** ***n*** (%)^*^			**Non-localizing** ***n*** (%)^*^		
	**REM**	**NREM**	**Awake**	**REM**	**NREM**	**Awake**	**REM**	**NREM**	**Awake**
(26)	4/6 (66.7)	3/6 (50)	n/a	2/6 (33.3)	3/6 (50)	n/a	0/6 (0)	0/6 (0)	n/a
(28)	15/20 (75)	3/20 (15)	10/20 (50)	0/20 (0)	0/20 (0)	0/20 (0)	5/20 (25)	17/20 (85)	10/20 (50)
(39)	10/11 (90.9)	9/11 (81.8)^a^	10/11 (90.9)	1/11 (9.1)	1/11 (9.1)^a^	1/11 (9.1)	0/11 (0)	1/11 (9.1)^a^	0/11 (0)
(29)	14/15 (93.3)	10/15 (66.7)	11/15 (73.3)	1/15 (6.7)	2/15 (13.3)	2/15 (13.3)	0/15 (0)	3/15 (20)	2/15 (13.3)
(19)	19/19 (100)	32/37 (86.5)	16/25 (64)	0/19 (0)	3/37 (8)	1/25 (4)	0/19 (0)	2/37 (5.4)	8/25 (32)
Percent average weighted per study (IQR)	85.2% (15.9)	60.0% (16.7)	69.6% (8.2)	9.8% (6.7)	16.1% (1.1)	6.6% (3.6)	5%	23.9% (3.7)	23.8% (12.7)

*n/a, not assessed*;

* *denominator based on number of patients with IEDs in that state (e.g., REM, NREM, awake)*.

a *NREM3 specifically*.

#### Quantitative Analysis

Out of five studies with a collective 91 patients in total, we found that REM IEDs provided a focal localization concurring with the clinical SOZ in 85.2% of patients, compared with NREM IEDs in 60.0% of patients, and awake IEDs in 69.6% of patients (Figure 5, Table 2). REM IEDs were rarely generalized (5.0% of patients) compared to other sleep-wake states (23.9% in NREM, 23.8% in wakefulness). Regarding false localization, REM IEDs provided a focal localization that was discordant with the clinical SOZ in 9.8% of patients, a rate similar to awake IEDs (6.6%) and less than NREM IEDs (16.6%).

### Interictal Localization by REM as Arbitrated by Post-resective Surgery Seizure Freedom

#### Literature Review

Next, we sought studies assessing the localizing value of REM as arbitrated by post-resective surgery seizure freedom, as this outcome retrospectively demonstrates complete resection of the EZ and is the gold standard for validating localization. From articles listed in Table 1, we identified four studies (collective 46 subjects) meeting criteria for analysis. Four other studies had included resective surgery, but one was excluded because it was a case report (41), one was excluded because the location of the resected zone was not reported (38), and two others were excluded because post-operative outcomes were unavailable for individual patients (28, 31).

For 3/4 studies, individual patient data were extracted straightforwardly from text, figures, and tables in the original manuscripts (23, 24, 29). Sakuraba et al. trichotomized electrodes into those displaying HFOs predominantly in REM, NREM, or neither by comparing z-scores of HFO occurrence rate (27). In order to extract individual patient data, we re-defined localization as the proportion of REM-predominant HFO electrodes inside vs. outside the zone of resection, and likewise for NREM-predominant HFO electrodes. For example, suppose a hypothetical patient has 100 total intracranial electrodes, 10 REM-predominant and 20 NREM-predominant. If >50% of REM-predominant electrodes lay inside the resected zone, we considered REM to have overall localized the resected zone. Likewise, if >50% of NREM-predominant electrodes lay inside the resected zone, we concluded NREM had also localized the resected zone (REM's and NREM's localizations not being mutually exclusive). Conversely, if >50% REM-predominant or NREM-predominant electrodes lay outside the zone of resection, we concluded that neither REM nor NREM had localized the resected zone.

#### Quantitative Analysis of REM

Across four studies, 27/46 patients went on to experience seizure freedom, retrospectively demonstrating complete resection of the EZ and validating pre-surgical localization. Amongst these 27 cases (with individual studies weighted equivalently, rather than individual patients between studies), we found that REM correctly localized the EZ 83.6% of the time (Figure 6, Table 3). Sixty-four percent of the time, REM localized the correct EZ in agreement with localizing data from other sleep-wake states. And 19.6% of the time, REM alone localized the EZ, unique among sleep-wake states.

**Table 3:** Interictal localization of REM arbitrated by post-resective surgery seizure freedom (Engel I outcome).

**References**	**Total Engel I patients (*n*)**	**Concordant with EZ**						**Discordant with EZ**								
		≥1 other SWS concordant^*^; ***n*** **(%)**			0 other SWS concordant^*^; ***n*** **(%)**			2 other SWS concordant^*^; ***n*** **(%)**			1 other SWS concordant^*^; ***n*** **(%)**			0 SWS concordant^*^; ***n*** **(%)**		
		**REM**	**NREM**	**Awake**	**REM**	**NREM**	**Awake**	**REM**	**NREM**	**Awake**	**REM**	**NREM**	**Awake**	**REM**	**NREM**	**Awake**
(27)^a^	**6**	2 (33.3)	2 (33.3)	n/a	2 (33.3)	1 (16.7)	n/a	0	0	n/a	1 (16.7)^b^	2 (33.3)	n/a	1 (16.7)	1 (16.7)	n/a
(29)	**8**	6 (75)	5 (62.5)	6 (75)	2 (25)^c^	0	0	0	3 (37.5)	0	0	0	2 (25)	0	0	0
(24)	**8**	7 (87.5)	n/a	n/a	0	n/a	n/a	0	n/a	n/a	1 (12.5)^d^	n/a	n/a	0	n/a	n/a
(23)	**5**	3 (60)	2 (40)	3 (60)	1 (20)	0	0	0	1 (20)	0	0	1 (20)	1 (20)	1 (20)	1 (20)	1 (20)
Percent average weight per study (IQR)		64% (24.8)	45.3% (14.6)	67.5% (7.5)	19.6% (12.1)	5.6% (8.4)	0%	0%	19.2% (18.8)	0%	7.3% (13.6)	17.8% (16.7)	22.5% (2.5)	9.2% (17.5)	12.2% (10)	10% (10)

*This is the gold-standard to validate EZ localization; concordance with EZ indicates correct localization. n/a, not assessed*;

* *denominator based on number of patients with IEDs in that state (e.g., REM, NREM, awake)*.

a *Wakefulness not analyzed*.

b *For this one patient, NREM concurred with EZ but REM contained insufficient quantity of interictal signals for localization*.

c *REM agree with side of ictal onset (n = 1) and largest tuber (n = 1)*.

d *In NREM and wakefulness, ictal localization was considered. IQR, interquartile range*.

However, in 16.5% of cases with post-operative seizure freedom, REM's localization was incorrect, localizing away from the resected region whose removal brought seizure freedom. In 9.2% of these, the localization data from REM and all sleep-wake states were incorrect (in such cases, IED localizations were discounted; resection was guided by other data). In 7.3%, REM's localization was incorrect, but a different sleep-wake state was used to guide resection and ultimately validated by the ensuing seizure freedom (Table 3). Interestingly, REM was never uniquely incorrect. In other words, if REM was incorrect, then at least one of NREM or wakefulness was also incorrect as well.

We further analyzed the localization of REM in patients who continued to have seizures post-operatively (Engel outcomes II–IV; Supplementary Table 1). These patients, for whom EZ localization was not validated, totaled 19 subjects across the same four studies. In 14.2% of cases, REM's localization uniquely concurred with the zone of resection (i.e., no other sleep-wake state concurred), suggesting erroneous localization by REM. In 14.6% of cases, REM's localization disagreed with the zone of resection, but another sleep-wake state guided resection. In these patients too, REM was never uniquely discordant with the resected zone; either NREM or wakefulness agreed with REM each time. In 56.7% of cases, REM and one or more other sleep-wake states concurred with the zone of resection. In 14.6% cases, all sleep-wake states disagreed with the zone of resection, and other data guided resection.

#### Quantitative Analyses of NREM and Wakefulness

For context and to avoid having analyzed REM in isolation, we also assessed the localizations of NREM and awake IEDs as validated by the gold-standard EZ in patients achieving post-operative seizure freedom. The same four studies in section Interictal localization by REM as Arbitrated by Post-resective Surgery Seizure Freedom were considered as pertinent; however, one was excluded because less localization data were reported for NREM and wakefulness than REM (24). Thus, for NREM, we extracted data from three studies and 19 subjects achieving post-operative seizure freedom. For analysis of localization in wakefulness, one further study was excluded because only REM and NREM were analyzed (27).

Out of three studies with 19/34 patients achieving post-operative seizure freedom, localization in NREM concurred with the gold-standard EZ 50.9% of the time, uniquely so in 5.6% of cases, and in conjunction with other sleep-wake states in 45.3% (Table 3). In the other 49.1%, NREM failed to localize the gold-standard EZ. In 36.9% of cases, NREM did not localize correctly, when one or more other sleep-wake states did. In 12.2% of cases, no sleep-wake state correctly localized the EZ, and other data guided resection.

In wakefulness, across two studies with 13/22 patients achieving post-operative seizure freedom, wakefulness correctly localized the gold-standard EZ 67.5% of the time, always in conjunction with one or more other sleep-wake states (Table 3). Wakefulness failed to localize the gold-standard EZ 32.5% of the time—of which 22.5% wakefulness failed, but one or more other states correctly found the EZ. Ten percent of the time, no sleep-wake state correctly found the EZ, and other data guided resection.

#### Subgroup Comparisons: Surface vs. Intracranial Recording; Localization vs. Lateralization

Next, using the same surgical studies, we performed a subgroup comparison of the localizing value of REM in surface vs. intracranial recordings, and in studies reporting lateralization vs. localization proper, arbitrated by the gold-standard EZ in both cases.

Regarding recording techniques, 2/4 studies used intracranial recording (23, 27) and 2/4 used surface recording (24, 29). When surface recording was used, REM concurred with the gold-standard EZ 93.8% of the time, vs. only 63.3% of the time when intracranial recording was used. Similarly, when surface recording was used, NREM and wakefulness concurred with the gold-standard EZ 62.5 and 75% of the time, respectively, vs. 45 and 60% when intracranial recording was used.

2/4 studies reported lateralization (23, 29) and 2/4 studies reported localization (24, 27). Wakefulness was excluded from study by Sakuraba et al. and Montplaisir et al., so for this subgroup comparison, we only considered REM and NREM. We found that REM concurred with the gold-standard EZ 80% of the time when lateralization was reported, vs. 77.1% of the time when localization was reported. NREM concurred with the gold-standard EZ 51.3% of the time when lateralization was reported, vs. 50% of the time when localization was reported.

### Effect on Post-operative Seizure Freedom of Including or Excluding Interictal Sleep-Wake State Localization in a Resection

Using the same 4 REM, 3 NREM, and 2 awake surgical studies from sections Interictal Localization by REM as Arbitrated by Post-resective Surgery Seizure Freedom and Effect on Post-operative Seizure Freedom of Including or Excluding Interictal Sleep-Wake State Localization in a Resection (23, 24, 27, 29), we also assessed the effect on seizure freedom of including the interictal localization as suggested by a sleep-wake state, and then compared this effect between sleep-wake states. For studies reporting lateralization rather than precise localization proper, we considered whether the resection occurred ipsilateral or contralateral to the lateralized hemisphere (23, 29). Weighting equivalently by study, when REM's localization was included in the resection, 59.7% of patients achieved seizure freedom, and 40.3% continued to suffer seizures (Table 4). When REM's localization was ignored in a resection, seizures continued in 50% of patients and were eradicated in 50% of patients. Results were similar for NREM and wakefulness. When NREM's localization was followed, 54.2% of patients achieved seizure freedom, and 45.8% continued to suffer seizures. When NREM's localization was ignored, seizures continued in 36.7% of patients and were eradicated in 63.3% of patients. When wakeful localization was followed, 67.5% of patients achieved seizure freedom, and 32.5% continued to suffer seizures. When wakeful localization was ignored, seizures continued in 25.0% of patients and were eradicated in 75.0% of patients.

**Table 4:** Effect on post-operative seizure freedom of including or excluding interictal sleep-wake state localization in resection.

**References**	**Localization included in resection**						**Localization excluded from resection**					
	**Seizure freedom** ***n*** **(%)**			**Ongoing seizures** ***n*** **(%)**			**Seizure freedom** ***n*** **(%)**			**Ongoing seizures** ***n*** **(%)**		
	**REM**	**NREM**	**Awake**	**REM**	**NREM**	**Awake**	**REM**	**NREM**	**Awake**	**REM**	**NREM**	**Awake**
(27)	3/6 (50)	3/6 (50)	n/a	3/6 (50)	3/6 (50)	n/a	3/6 (50)	3/6 (50)	n/a	3/6 (50)	3/6 (50)	n/a
(29)	8/13 (61.5)	5/8 (62.5)	6/8 (75)	5/13 (38.5)	3/8 (37.5)	2/8 (25)	n/a	0/1 (0)	0/2 (0)	n/a	1/1 (100)	2/2 (100)
(24)	7/10 (70)	n/a	n/a	3/10 (30)	n/a	n/a	1/2 (50)	n/a	n/a	1/2 (50)	n/a	n/a
(23)	4/7 (57.1)	2/4 (50)^a^	3/5 (60)	3/7 (42.9)	2/4 (50)	2/5 (40)	1/2 (50)	3/5 (60)	2/4 (50)	1/2 (50)	2/5 (40)	2/4 (50)
Percent average weighted per study (IQR)	59.7% (8.3)	54.2% (6.3)	67.5% (7.5)	40.3% (8.3)	45.8% (6.3)	32.5% (7.5)	50% (0)	36.7% (30)	25% (25)	50% (0)	63.3% (30)	75% (25)

*We excluded patients in whom IEDs were present but non-localizing (e.g., generalized). For studies reporting lateralization rather than precise localization proper, we considered whether the resection occurred ipsilateral or contralateral to the lateralized hemisphere*.

a *Original authors report light sleep and deep sleep both concordant (n = 3) and light sleep concordant but deep sleep was discordant (n = 1). IQR, interquartile range*.

### REM's Effects on IED Field Size

#### Literature Review

In light of the possible localizing value of REM and certain mechanistic arguments involving cortical synchrony, we next sought work assessing REM's effects on the absolute field size of interictal epileptic phenomena. Of 19 pertinent studies in Table 1, five (including one case report) contained report of REM altering the absolute field size of interictal epileptic phenomena (Figure 4B). Of these, three met criteria for analysis. One was excluded because it was a case report (41), and another did not provide per-patient data (34).

#### Quantitative Analysis

Studies and methods were heterogeneous, so we will briefly describe them. Kang et al. report a 53% reduction in scalp IED field size in REM compared to NREM, and, using source reconstruction techniques, a 24% size reduction in the spatial extent of probabilistic source generators (26). Okanari et al. report that, amongst 20 patients with generalized IEDs yet non-lesional MRIs, REM lateralized the IED scalp field in 15 (28). Ng reports that, of 69 EMU patients, 39 of whom had IEDs in REM (43), REM IEDs clarified the localization in seven (37). In 6/7 patients, IED field size was clearly reduced relative to localization data from other sleep-wake states. Weighting equivalently by study, REM shrinks the IED field in 63.5% of cases (Figure 7, Supplementary Table 2).

### REM's Effects on Spatial Novelty of the IED Field

#### Literature Review

Next, we sought literature on the possibility that REM could activate interictal epileptic phenomena in unique spatial coordinates. Of 19 studies in Table 1, 4 original works (including one case study) report REM's IED field encompassing novel spatial coordinates neglected by other sleep-wake states (Figure 4C). Studies were heterogeneous, so we will briefly describe them.

#### Quantitative Analysis

In Kang et al.'s recent report on spatial characteristics of REM vs. NREM IEDs, REM surface IEDs appeared to occupy new spatial territory relative to NREM IEDs in 4/6 patients (26). This assessment was made on our visual inspection of scalp IED fields in an original figure. Amongst 3/6 patients with epilepsy, Watanabe et al. report that REM IEDs occupied novel spatial fields (40). In Genton et al.'s case report of Landau-Kleffner Syndrome, REM IEDs propagated bilaterally, uniquely so among sleep-wake states (32). Mayersdorf and Wilder studied 9 patients with temporal lobe seizures and found that in 1 patient, REM IEDs originated from a novel focus (25). Given a high number of case reports and overall diverse methodologies, we simply compiled these works and enumerated the fraction of patients in whom the REM IED field revealed new spatial coordinates (Figure 8).

### Pertinent Literature Excluded From Quantitative Analyses

Six studies were pertinent to spatial and/or localizing performance of REM, but were excluded from our quantitative analyses because they did not report per-patient data for majority of subjects (21, 31, 34, 38) or because they were case studies (18, 42).

In 30 subjects with medically-refractory focal epilepsy who underwent resective surgery, Klimes et al. analyzed pre-surgical iEEG recordings with a support vector machine learning model to retrospectively predict stereo-EEG contacts within the zone of resection (31). It was found that NREM2 and NREM3 recordings contained features to best localize the EZ. Although per-patient post-operative outcome data was unavailable, 13/30 subjects achieved seizure freedom. Scarlatelli-Lima et al. studied 56 patients with medically-refractory mesial temporal lobe epilepsy (MTLE) and report that, of 12 patients with bilateral NREM IEDs, 5 had unilateral IEDs in REM (38). In 4 patients, unilateral REM IEDs concurred with a unilateral lesion on MRI. Singh et al. report in subjects with medically-refractory focal epilepsy that NREM scalp IEDs were of highest localizing yield (21). 12 patients had a “different population” of REM IEDs, concordant with other localizing data in 7 patients, and discordant in 5 patients. Beyond this, localizing data from REM were unavailable. Von Ellenrieder et al. demonstrate using iEEG that pathological ripples are less spatially extensive in REM than in NREM-2 and −3 (awake and NREM-1 not assessed) (34). Spatial extent data per patient were unavailable. Malow et al. report that, in 21 patients with medically-refractory MTLE, only 1 subject had bilateral IEDs in REM, whereas 10 subjects did in NREM (18). This may suggest that REM IEDs were less spatially extensive; however, a unilateral IED is not necessarily smaller than a bilateral one (see section Heterogeneous Measures of REM IED Spatial Location: Lateralization vs. localization, structural vs. functional bounds), and localization was not arbitrated by EZ or SOZ. Bazil and Walczak report that focal onset seizures are less likely to generalize in REM than in NREM or wakefulness (42).

## Discussion

### REM Can Make IED Fields Smaller: Good but Not Perfect

Here we demonstrate among existing literature that REM generally performs well at localizing the SOZ and EZ. Across studies comparing IED localization with clinical SOZ, IEDs in REM concur with the clinical SOZ in 85.2% of cases, more than any other sleep-wake state. Across studies validating EZ localization with post-resective surgery seizure freedom, REM concurred with the zone of resection in 83.6% of cases, more than any other sleep-wake state. These results suggest a vital role of REM in localization.

Mechanistically, REM may aid interictal localization by constraining propagation of IEDs to a restricted spatial zone due largely to cortical asynchrony (44–46). The observed cortical asynchronous EEG pattern in REM may relate to asynchronous, single-spike firing displayed by the thalamus (47). Thalamic shift from rhythmic burst generation to asynchronous single-spike firing occurs in REM (48). To initiate REM, ascending “REM-on” networks increase cholinergic input to the thalamus (48, 49). The activity of REM-on circuitry in turn appears gated by orexinergic signaling (50), such that when orexin tone drops, REM-on networks activate, and REM begins. The resulting cortical asynchrony ensures a lack of endogenous coordinated activity to propagate epileptic phenomena. As a result, REM IEDs remain comparatively local to the epileptogenic foci.

In contrast, highly coordinated activity in NREM is thought to easily propagate IEDs (51). One clear example is the phenomenon of “dyshormia,” which refers to IEDs that propagate by mounting physiologic K-complexes (52). Of NREM stages, slow-wave sleep appears to have the strongest facilitative effect on epileptic activity (53). Heightened synchrony in slow-wave sleep largely manifests in namesake slow-waves (49), comprised of cyclically alternating “up” and “down” components (51, 54, 55). Frauscher et al. recently demonstrated that, within slow-waves, it is the transition from up- to down-state—a particularly synchronized part of the cycle—that contains most IEDs and HFOs (53). Thus, cortical synchrony seems to render NREM permissive to facilitate and propagate IEDs, perhaps especially in highly-synchronous up-down cyclic alternating patterns (CAP) of slow-wave NREM. Conversely, absence of highly synchronous microfluctuations in REM, and REM's overall asynchrony, may suppress IEDs and improve localization.

In reports where such data was available, REM shrank the absolute IED field size in 63.5% of patients. These reports may be subject to publication bias (discussed in section Limitations). Other work did not meet criteria for analysis but were nonetheless supportive; for example, pathological REM ripples demonstrably occupy a smaller spatial field than in NREM-2 and−3 on iEEG (34). Amongst Malow et al.'s seven patients with REM IEDs, only 1 had bilateral REM IEDs, compared to 10 patients with bilateral NREM IEDs (18). Similarly, Malow and Aldrich report a case in which NREM lateralized to both left and right temporal regions, and ictal EEG showed independent bilateral ictal onset, but all REM IEDs lateralized to the left temporal region (41). REM IEDs narrowed localization and helped guide resective surgery, which brought seizure freedom for the duration of reported follow-up. Scarlatelli-Lima et al. report that REM IEDs lateralized in 5/12 patients with bilateral NREM IEDs, and lateralized concordantly with side of MRI lesion in 4/5 (38). These works suggest valuable localization by REM, in part by reducing the spatial field. However, it is imperative that absolute spatial attributes not be conflated with clinically-relevant localization, as a unilateral IED field is not necessarily smaller than a bilateral one (see section Heterogeneous Measures of REM IED Spatial Location: Lateralization vs. Localization, Structural vs. Functional Bounds).

While most literature we obtained focused on interictal epileptic phenomena in REM, Bazil and Walczak captured focal seizures in REM (5), NREM (189), and wakefulness (428) (42). Of these, 0 seizures in REM generalized, compared to 67 (35.4%) in NREM and 77 (18.0%) in wakefulness. This result awaits reproduction, especially with a large sample of REM seizures. Nonetheless, it is intriguing to speculate that REM's asynchronous milieu may suppress propagation of ictal activity. While IEDs and seizures are both rare in REM sleep (22), seizures appear rarer than IEDs. It is thought that this may be due to the higher threshold of neuronal synchrony that must be achieved for ictal commencement (50), given that seizures are highly synchronous events (56). Accordingly, when aberrant neuronal activity musters sufficient synchrony to overcome REM and break through as a seizure or IED, this may portend worse clinical prognosis (43). Altogether, REM's asynchrony may exert endogenous suppressive effects on both occurrence and spatial extent of interictal and ictal phenomena.

Comparing REM and wakefulness in particular, while REM appears superior in terms of interictal SOZ and EZ localization, we interestingly found relatively fewer patients with seizure freedom following a resection that included REM's interictal localization compared to patients whose resection included wakefulness's interictal localization. Furthermore, when REM's interictal localization was excluded, fewer patients fared poorer than when wakefulness' interictal localization was excluded. Considering the caveat that resection heeds far more data than simply sleep-wake interictal localization, these post-operative results may be explained in part by asynchrony, as wakefulness can exhibit similar asynchrony on EEG as REM (though achieved through a different mechanism). It is possible that asynchrony in wakefulness may allow a degree of localization that is similar or superior to REM in some respects, which seems reflected in our finding that including or excluding wakefulness' interictal localization may affect post-operative seizure freedom following a resection. Whether REM has an asynchronous advantage over wakefulness remains an open question, and further study is needed to better illuminate these intriguing prospects.

### REM Can Find New IED Field Coordinates: Good but Not Perfect

REM may also introduce novel IED field coordinates not encompassed by IEDs in other states. In the literature we found numerous, heterogeneous such reports. This result may account for the ability of REM to attain a localization that is uniquely correct among sleep-wake states, such as patients achieving post-resective surgery seizure freedom following resection of an EZ that was localized uniquely by REM. Overall, we found that in 19.6% of cases, REM uniquely revealed a true EZ when other sleep-wake states could not. Although the mechanism of REM's unique localizing ability remains to be fully characterized, it is thought that REM's suppressive effects may be of lesser magnitude on epileptogenic cortex compared to non-epileptogenic cortex (27, 45), thus revealing true epileptogenic foci. Specifically, REM has been shown to markedly decrease HFOs in the irritative zone, but was far less able to decrease HFOs in the EZ (27). The mechanisms underlying these phenomena are unclear; future study is needed to explore this intriguing result.

However, REM's unique localization may also be wrong, such as in patients achieving seizure freedom following resection of an EZ to which REM did not localize, and in patients for whom REM-guided EZ resection did not bring seizure freedom. In our review, we found that in 7.3% of cases, REM was wrong when other sleep-wake states correctly localized a true EZ. But when REM was wrong, it was never uniquely wrong by incorrectly localizing the EZ on its own despite correct localizations of both NREM and wakefulness. In other words, each time REM was wrong, so was NREM and/or wakefulness. Interestingly, the same holds true for wakefulness with regards to REM and NREM, which may further support asynchrony being a mechanism for unique localizing abilities given that wakefulness can demonstrate similar asynchrony as REM on EEG. Likewise in considering absolute IED field size, whether REM has an asynchronous advantage over wakefulness remains an open question. If so, then this may explain REM's comparable unique localizing advantage over wakefulness, as no studies in our review demonstrated that wakefulness could uniquely reveal the true EZ when other sleep states could not, while REM did so in 20%.

Nevertheless, it is generally difficult to draw inferences from localization data of patients who continue to experience seizures following resective surgery, as patients may continue to seize due to preservation of eloquent cortex, technical surgical difficulties, or other reasons. For example, Okanari et al. report that, of 4 of 17 patients who continued to have seizures post-operatively, 1 patient had undergone purposefully incomplete resection of the putative EZ to preserve eloquent cortex (28). Outside the surgical setting, REM's novel spatial data has also occasionally localized away from any apparent veridical SOZ or EZ, such as in Genton et al.'s case report in which REM IEDs spread extensively and bilaterally (32). It is unclear why REM may promote extensive IED spread. One hypothesis is that REM's cortical asynchrony may occasionally cause paradoxical constructive interference leading to diffuse propagation of IEDs. Another possibility is that there is something intrinsically different about REM in these individuals that facilitates localization spread.

Regardless of underlying biological mechanism, the introduction of novel spatial coordinates by REM can either enhance or diminish localizing value, and this cannot be deduced from spatial novelty alone. Watanbe et al. report that, for 3 of their 4 patients with REM IEDs, REM IEDs occupied novel spatial fields (40). In one of Mayersdorf and Wilder's patients, REM IEDs originated from a novel focus (25). For each patient, it is impossible to know whether the unique spatial territory covered by REM IEDs increases or decreases accuracy of localization without considering additional clinical data.

### Heterogeneous Measures of REM IED Spatial Location: Lateralization vs. Localization, Structural vs. Functional Bounds

Regarding metrics of localization, studies included in our analyses were heterogeneous. Rather than localizing a specific three-dimensional spatial region, many simply considered lateralization to the epileptic hemisphere (18, 19, 23, 28, 38, 39). Of these, some were conducted in patients with MTLE (19, 38, 39) or tuberous sclerosis complex (TSC) (28), where MRI-visible lesions provide additional means of further triangulating epileptogenic lesions within the hemisphere. Only two studies considered specific anatomical *local*-ization, rather than *lateral*-ization, in the absence of a constraining anatomical boundary such as a hemisphere or a lobe (26, 27).

Sakuraba et al. spatially resolved individual intracranial electrodes and classified them as inside or outside the zone of resection (27). Kang et al. performed current density reconstruction from surface EEG to assess lobar correspondence with clinical SOZ (26). Both compared REM vs. NREM, but did not assess wakefulness. Each report REM to be of superior localizing value than NREM.

We performed a subgroup comparison of REM's localization performance vis-à-vis lateralization vs. localization and did not find a significant difference. However, this comparison was limited by small number of studies and patients. Additionally, comparing lateralization vs. localization retrospectively among existing works may present a false dichotomy. Studies reporting mere lateralization likely would have noted additional localizing data for use in clinical care, especially pre-surgical planning; but since this data was not reported in the articles, it may confound certain results.

Lateralization should be more commonly achieved than true localization, as lateralization has a limited set of possibilities (ipsilateral, contralateral, bilateral). Even if localizing with only *lobar* specificity (i.e., localizing to a particular cerebral lobe and not any more finely than that), there are at least eight distinct possibilities (e.g., left or right frontal, parietal, temporal, occipital) with some overlap possible and not including the insulae. Lateralization may also be relatively easier to achieve by the relative anatomical divide between hemispheres obstructing IED spread, rendering it more likely that a discharge will remain confined to the hemisphere in which it arose. In contrast, there is far less of an anatomical divide between lobes, which are relatively bounded more on functional than anatomic grounds.

One can zoom in further and move from “lobar” localization to a theoretical source “point” localization of the so-called epileptic generator (Figure 2). In reconstructing the intracranial source generator from surface EEG signals—either formally via MN imaging or LORETA, or implicitly in clinical localization—the statistical bound of probable source generators may or may not concur perfectly with any one cerebral lobe. Therefore, if the probability density of IED source generators reproducibly and precisely localizes to a particular spatial region that is not demarcated by an anatomical bound, then this may be more suggestive of finding the true EZ, because the bounds of this probability density are not dependent on a well-known and easily demarcated anatomical bound (be it inter-hemispheric or inter-lobar). The reliability of EZ ascertainment is even higher if it is concordant with a putative lesion established independently by other means that had not factored into the source localization analysis itself (such as MRI).

Altogether, future studies assessing the localization of REM absent a confining anatomical bound will be a true test of its localizing performance.

### Other Sources of Variation: Surface vs. Intracranial Recording, and Biological Heterogeneity

We also assessed whether surface vs. intracranial recording techniques affected the localizing performance of REM and other sleep-wake states. Arbitrated by gold standard EZ, the localizing abilities of REM, NREM, and wakefulness each appeared worse amongst studies invasively recording. This may be due to sampling bias and imperfect cortical coverage, which can lead to important epileptic phenomena being missed, artificially diminishing localization yield. There is also a selection bias for intracranial recording in patients with epilepsy that is more difficult to localize on surface recording, which would be expected to impact the accuracy of the putative EZ suggested by a sleep-wake state from surface recording as well. Further work is needed to assess whether the localization of REM might fare better or worse in a rigorous prospective study comparing surface vs. intracranial recordings. Intracranial recordings also present the opportunity to capture different types of signal; for example, HFOs being more commonly captured intracranially than at scalp level (27, 31, 34). It remains to be seen if localizing based on different types of signals has any effect on REM's localizing performance.

As an alternative to invasive intracranial recording, sophisticated source reconstruction techniques allow estimation of cortical sources from non-invasive scalp recording (26). Source reconstruction methods such as standardized LORETA assign a probabilistic current vector to each brain voxel (57). Thus, to define a source location, a statistical bound must be set, with 50% of the maximum statistical likelihood (known as “full-width at half-maximum”) a commonly used threshold (58, 59). Accuracy of probabilistic source location is typically improved by higher density of electrodes. However, high density EEG is generally not in widespread clinical use, due to laborious set-up and analysis. Studies using high-density EEG studies might therefore understandably utilize a smaller sample size, leading to trade-off between accuracy of source reconstruction via high density of electrodes vs. greater sample size and higher per-patient statistical power in clinical settings using routine EEG.

Biological heterogeneity is another source of variability that may also affect our results. Studies analyzed were demographically heterogeneous. Most studies used adult subjects, but we also included studies with pediatric subjects (28, 29). Different disease processes also introduce heterogeneity. Due to a small number of studies and non-standardized reporting of patient clinical characteristics, we were unable to assess whether REM localizes better or worse in MTLE, neocortical TLE, TSC, or in other specific diseases. It is also possible that long disease duration may affect localization, as epileptic foci can enlarge over time, but disease duration was not reported by most authors. Although Okanari et al. reported disease duration, this may be a less standardized metric in their pediatric population due to individualized cerebral maturational changes and an upper bound of disease duration set by birth, given that patients ranged from 1 to 17 years of age (28). In the study of Rocamora et al. where disease duration was also reported, when one or more sleep-wake state did not lateralize to the putative SOZ, patients tended to have longer disease duration (39).

### Dénouement: Incorporating REM IED Field Size and Location into a Set-Theoretic Model of Concordance

Discussions of spatial attributes of epileptic phenomena in REM in the literature usually suffer from certain recurrent ambiguities. For example, lateralization does not necessarily convey information about extent of spatial field. IEDs may occupy a small but bilateral spatial field, whereas a unilateral IED could occupy an entire hemisphere. Thus, REM may lateralize an IED field without necessarily reducing its absolute size. Even when an IED field is made smaller, this might not translate to an accurate localization of the EZ. A larger field may be more accurate than a smaller field; for example, if the larger one includes some or all of the EZ when the smaller one does not. In other words, smaller REM IED field size is only useful when it is concordant with the location of the EZ.

**Figure 9:**
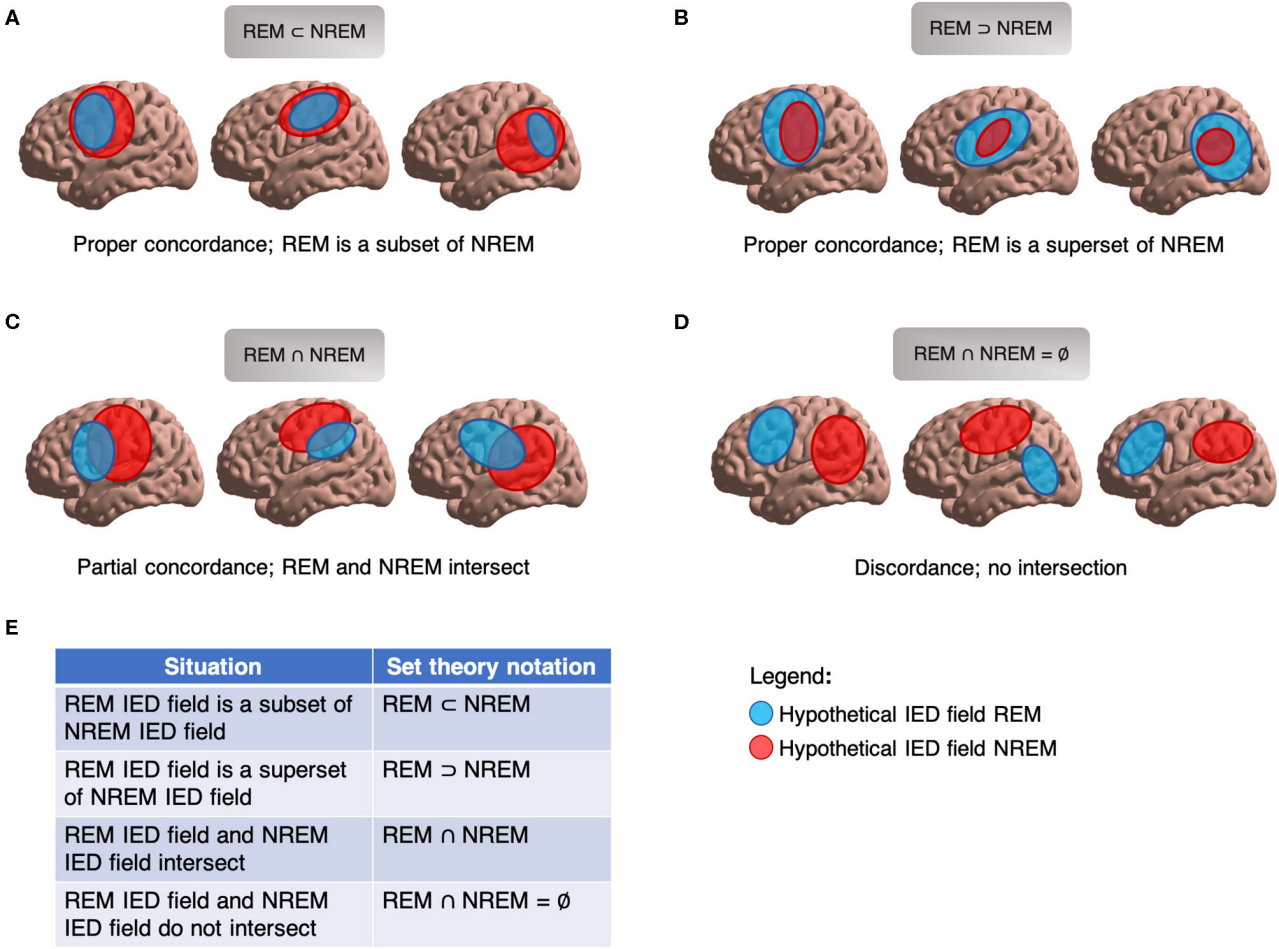
Set theory for IED fields. **(A)** Proper concordance, NREM IED field subsumes REM IED field. **(B)** Proper concordance, REM IED field subsumes NREM IED field. **(C)** Partial concordance; REM and NREM IED fields overlap. **(D)** Discordance; REM and REM IED fields do not overlap. **(E)** Set theory notation for describing relationships of IED fields.

To clearly and efficiently depict these spatial size-locational relations between IED fields, we suggest adopting a simple mathematical set-theoretic framework to formalize descriptions of spatial concordance and discordance (Figure 9); specifically, those of “proper concordance,” “partial concordance,” and “discordance.” In proper concordance, one field engulfs another field (i.e., one coordinate set is a subset of another coordinate set). For example, the IED field of REM may localize more specifically than in wakefulness, but wholly within a sub-region of the awake IED field. Essentially, REM narrows the field and does not provide any unique spatial information. The converse is also possible in that a different sleep-wake state may localize to a specific small region entirely subsumed by the localization of REM's IED field. In partial concordance, two IED fields overlap but neither entirely subsumes the other (Figure 9B). Each provides some unique spatial data. In practice, where there is relatively little anatomic bound between lobes, partial concordance is likely. Partial concordance can be quantified, such as by percent spatial overlap. In discordance, two IED fields do not overlap, sharing no spatial coordinates (Figure 9C). If desired, formal set theory notation could even be used, such as the overlap metric “2^*^(A∩B)/(A+B),” where A and B are sets representing different sleep-wake IED fields. This metric can be easily applied to proper concordance, partial concordance, and discordance to help facilitate streamlined analysis and discussion of sleep-wake spatial localization in future studies.

Also importantly, these spatial considerations are agnostic to clinical anchors such as SOZ or EZ. Two properly-concordant IED fields may concur with a false localization. Likewise, if confronted with two discordant IED fields, additional data are required to know which (if either) field provides the correct localization. These spatial considerations also do not hinge on localization by imaging studies. For example in unifocal epilepsy, a brain imaging study may disclose a non-localizing (or non-specifically localizing) focus, a localizing focus discordant to the EZ, a localizing focus concordant to the EZ, or no focus at all. A conceptually clear set-theoretic terminology for describing IEDs and other localizing phenomena in space will help guide future investigation into important questions of REM's ability to triangulate epileptic foci by providing a clean classification framework within which to intuitively and simultaneously convey new findings on size, location, and concordance. Such a framework may also be applied to ictal phenomena, other sleep-wake states aside from REM, and to any general situation dealing with source localization.

## Limitations

Our quantitative analyses were limited due to a small number of studies, some with small sample sizes. Due to a lack of per-patient data, we had to exclude certain otherwise pertinent studies from quantitative analyses. Among studies quantitatively analyzed, methodologies were heterogeneous, limiting the precise numerical validity of some of our results and precluding the possibility of a rigorous statistical meta-analysis. Heterogeneous approaches and terminologies also occasionally required re-interpretation of originally reported data to extract the pertinent spatial and localizing information required for our study. Given the need for re-interpretation, it is impossible to rule out the inadvertent exclusion of a few tangentially-pertinent articles with data potentially amenable to substantial re-interpretation; however, to mitigate this, we assigned multiple authors to review the literature and enhance objectivity. Of studies included in our review, most reported only lateralization, not localization *per se*. Lateralization is generally the least specific method of spatially triangulating the EZ (Figure 2) and is thus a less rigorous measure of assessing localizing performance. There is also a possibility of publication bias in favor of preferentially reporting instances in which REM was clinically useful, especially in case studies and other works with small sample size.

Where quantitative analysis was possible, we chose to weight each study equivalently rather than each patient equivalently. This has the upside of preserving the signal of certain recent works with small sample size but rigorous methodology (26); however, it may also generally over-weight findings from smaller studies. Further, comparisons between sleep-wake states may be unfair when there are different amounts of studies for different states, e.g., REM (4 studies) vs. NREM (3 studies) vs. wakefulness (2 studies) in localizing the EZ as arbitrated by post-operative seizure freedom, and in predicting post-operative seizure freedom. Nonetheless, despite these limitations and to the best of our knowledge, this is the first work to systematically characterize across multiple studies the important intriguing question of the localizing value and spatial attributes of epileptic phenomena in REM. In general, methods to improve the yield of interictal localization remain urgently needed, and we hope to further illuminate REM's important role.

## Conclusion

In this paper, we have systematically reviewed, and quantitatively analyzed whenever possible, the literature on REM's ability to localize the SOZ and EZ in human epilepsy patients. We found that REM's localization outperformed that of any other sleep-wake state, and that, in some patients, REM can shrink the IED field and/or introduce new spatial coordinates to the IED field to render a unique localization that is often more helpful than not. In our extensive review, we were often confronted with the fact that the localizing value of changes in IED field size and location can only be truly assessed by incorporating further considerations aimed at delineating EZ concordance. Therefore, to accurately and efficiently describe surprisingly tricky spatial relations between two IED fields, such as those from two sleep-wake states, we introduce a simple, easy-to-use, and conceptually clear set-theoretic framework. We hope that adoption of this framework will streamline and accelerate future discussion and research on the basic mechanisms and clinical applications of the fascinating anti-epileptic properties of REM.

## Data Availability Statement

All datasets presented in this study are included in the article/Supplementary Material.

## Author Contributions

AG and MN conceived the study. GM, AG, and MN designed the study. GM and MN performed the literature review, revised the manuscript, and read and approved the final version. GM performed the analysis and prepared the first draft of the manuscript. All authors contributed to the article and approved the submitted version.

## Conflict of Interest

The authors declare that the research was conducted in the absence of any commercial or financial relationships that could be construed as a potential conflict of interest.

## References

[1] England MJ Liverman CT Schultz AN Strawbridge LM. Summary: a reprint from epilepsy across the spectrum: promoting health and understanding. Epilepsy Curr Am Epilepsy Soc. (2012) 12:245–53. 10.5698/1535-7511-12.6.245 23447726 PMC3577135

[2] Devinsky O Spruill T Thurman D Friedman F. Recognizing and preventing epilepsy-related mortality: a call for action. Neurology. (2016) 86:779–86. 10.1212/WNL.0000000000002253 26674330 PMC4763802

[3] Feigin VL Nichols E Alam T Bannick MS Beghi E Blake N Global, regional, and national burden of neurological disorders, 1990–2016: a systematic analysis for the global burden of disease study 2016. Lancet Neurol. (2019) 18:459–80. 10.1016/S1474-4422(18)30499-X 30879893 PMC6459001

[4] Kwan P Sander JW. The natural history of epilepsy: an epidemioloqical view. J Neurol Neurosurg Psychiatry. (2004) 75:1376–81. 10.1136/jnnp.2004.045690 15377680 PMC1738749

[5] Kwan P Brodie MJ. Early identification of refractory epilepsy. N Engl J Med. (2000) 342:314–9. 10.1056/NEJM200002033420503 10660394

[6] de Tisi J Bell GS Peacock JL McEvoy AW Harkness WF Sander JW. The long-term outcome of adult epilepsy surgery, patterns of seizure remission, and relapse: a cohort study. Lancet. (2011) 378:1388–95. 10.1016/S0140-6736(11)60890-8 22000136

[7] Picot MC Jaussent A Neveu D Kahane P Crespel A Gelisse P. Cost-effectiveness analysis of epilepsy surgery in a controlled cohort of adult patients with intractable partial epilepsy: a 5-year follow-up study. Epilepsia. (2016) 57:1669–79. 10.1111/epi.13492 27595433

[8] Langfitt JT Holloway RG McDermott MP Messing S Sarosky K Berg AT. Health care costs decline after successful epilepsy surgery. Neurology. (2007) 68:1290–8. 10.1212/01.wnl.0000259550.87773.3d 17438219

[9] Lüders HO Textbook of Epilepsy Surgery. London: CRC Press (2008). 10.3109/9780203091708

[10] Rosenow F Lüders H. Presurgical evaluation of epilepsy. Brain. (2001) 124:1683–700. 10.1093/brain/124.9.1683 11522572

[11] Javidan M. Electroencephalography in mesial temporal lobe epilepsy: a review. Epilepsy Res Treat. (2012) 2012:1–17. 10.1155/2012/637430 22957235 PMC3420622

[12] Michel CM He B EEG mapping and source imaging. In: Schomer DL Lopes da Silva FH editors. Niedermeyer's Electroencephalography: Basic Principles, Clinical Applications, and Related Fields, Vol. 1 New York, NY: Oxford University Press (2017). p. 1135–56. 10.1093/med/9780190228484.003.0045

[13] Gibbs EL Gibbs FA Diagnostic and localizing value of electroencephalographic studies in sleep. J Nervous Mental Dis. (1947) 26:336–76.

[14] Passouant P Historical views on sleep and epilepsy. In: Sterman MB editor. Sleep and Epilepsy. New York, NY: Academic Press (1982). p. 1–6.

[15] Halász P. How sleep activates epileptic networks? Epilepsy Res Treat. (2013) 2013:1–19. 10.1155/2013/425697 24159386 PMC3789502

[16] Khan S Nobili L Khatami R Loddenkemper T Cajochen C Dijk DJ. Circadian rhythm and epilepsy. Lancet Neurol. (2018) 17:1098–108. 10.1016/S1474-4422(18)30335-1 30366868

[17] Frauscher B Gotman J. Sleep, oscillations, interictal discharges, and seizures in human focal epilepsy. Neurobiol Dis. (2019) 127:545–53. 10.1016/j.nbd.2019.04.007 30981828

[18] Malow BA Lin X Kushwaha R Aldrich MS. Interictal spiking increases with sleep depth in temporal lobe epilepsy. Epilepsia. (1998) 39:1309–16. 10.1111/j.1528-1157.1998.tb01329.x 9860066

[19] Sammaritano M Gigli GL Gotman J. Interictal spiking during wakefulness and sleep and the localization of foci in temporal lobe epilepsy. Neurology. (1991) 41(2 Pt 1):290–7. 10.1212/WNL.41.2_Part_1.290 1992379

[20] Frost JD Jr Hrachovy RA Glaze DG McCully MI. Sleep modulation of interictal spike configuration in untreated children with partial seizures. Epilepsia. (1991) 32:341–6. 10.1111/j.1528-1157.1991.tb04661.x 1904344

[21] Singh S Shukla G Goyal V Srivastava AK Singh MB Vibha D. Impact of sleep on the localizing value of video EEG in patients with refractory focal seizures - a prospective video-EEG with EOG and submental EMG study. Clin Neurophysiol. (2014) 125:2337–43. 10.1016/j.clinph.2014.03.021 24856459

[22] Ng M Pavlova M. Why are seizures rare in rapid eye movement sleep? Review of the frequency of seizures in different sleep stages. Epilepsy Res Treat. (2013) 2013:1–10. 10.1155/2013/932790 23853720 PMC3703322

[23] Lieb JP Joseph JP Engel J Jr Walker J Crandall PH. Sleep state and seizure foci related to depth spike activity in patients with temporal lobe epilepsy. Electroencephalogr Clin Neurophysiol. (1980) 49:538–57. 10.1016/0013-4694(80)90396-X 6158435

[24] Montplaisir J Laverdière M Saint-Hilaire JM Rouleau I. Nocturnal sleep recording in partial epilepsy: a study with depth electrodes. J Clin Neurophysiol. (1987) 4:383–8. 10.1097/00004691-198710000-00003 3119661

[25] Mayersdorf A Wilder BJ Focal epileptic discharges during all night sleep studies. Clin Electroencephalogr. (1974) 5:73–87. 10.1177/155005947400500203

[26] Kang X Boly M Findlay G Jones B Gjini K Maganti R. Quantitative spatio-temporal characterization of epileptic spikes using high density EEG: differences between NREM sleep and REM sleep. Sci Rep. (2020) 10:1673. 10.1038/s41598-020-58612-4 32015406 PMC6997449

[27] Sakuraba R Iwasaki M Okumura E Jin K Kakisaka Y Kato K. High frequency oscillations are less frequent but more specific to epileptogenicity during rapid eye movement sleep. Clin Neurophysiol. (2015) 127:179–86. 10.1016/j.clinph.2015.05.019 26073183

[28] Okanari K Baba S Otsubo H Widjaja E Sakuma S Go CY. Rapid eye movement sleep reveals epileptogenic spikes for resective surgery in children with generalized interictal discharges. Epilepsia. (2015) 56:1445–53. 10.1111/epi.13081 26174651

[29] Ochi A Hung R Weiss S Widjaja E To T Nawa Y. Lateralized interictal epileptiform discharges during rapid eye movement sleep correlate with epileptogenic hemisphere in children with intractable epilepsy secondary to tuberous sclerosis complex. Epilepsia. (2011) 52:1986–94. 10.1111/j.1528-1167.2011.03198.x 21801167

[30] Bagshaw AP Jacobs J LeVan P Dubeau F Gotman J. Effect of sleep stage on interictal high-frequency oscillations recorded from depth macroelectrodes in patients with focal epilepsy. Epilepsia. (2009) 50:617–28. 10.1111/j.1528-1167.2008.01784.x 18801037 PMC3792080

[31] Klimes P Cimbalnik J Brazdil M Hall J Dubeau F Gotman J. NREM sleep is the state of vigilance that best identifies the epileptogenic zone in the interictal electroencephalogram. Epilepsia. (2019) 60:2404–15. 10.1111/epi.16377 31705527

[32] Genton P Maton B Ogihara M Samoggia G Guerrini R Medina MT. Continuous focal spikes during REM sleep in a case of acquired aphasia (Landau-Kleffner syndrome). Sleep. (1992) 15:454–60. 10.1093/sleep/15.5.454 1280854

[33] Levenstein D Watson BO Rinzel J Buzsáki G. Sleep regulation of the distribution of cortical firing rates. Curr Opin Neurobiol. (2017) 44:34–42. 10.1016/j.conb.2017.02.013 28288386 PMC5511069

[34] von Ellenrieder N Dubeau F Gotman J Frauscher B. Physiological and pathological high-frequency oscillations have distinct sleep-homeostatic properties. NeuroImage Clin. (2017) 14:566–73. 10.1016/j.nicl.2017.02.018 28337411 PMC5349616

[35] Wieser HG Blume WT Fish D Goldensohn E Hufnagel E King D. Proposal for a new classification of outcome with respect to epileptic seizures following epilepsy surgery. Epilepsia. (2008) 42:282–6. 10.1046/j.1528-1157.2001.4220282.x 11240604

[36] Engel J Surgical Treatment of the Epilepsies. New York, NY: Raven Press (1993). p. 786.

[37] Ng MC. Maximizing the yield of rapid eye movement sleep in the epilepsy monitoring unit. J Clin Neurophysiol. (2017) 34:61–4. 10.1097/WNP.0000000000000312 27490323

[38] Scarlatelli-Lima AV Sukys-Claudino L Watanabe N Guarnieri R Wal R Lin K. How do people with drug-resistant mesial temporal lobe epilepsy sleep? A clinical and video-EEG with EOG and submental EMG for sleep staging study. eNeurologicalSci. (2016) 4:34–41. 10.1016/j.ensci.2016.06.002 29430547 PMC5803108

[39] Rocamora R Andrzejak RG Jimenez-Conde J Elger CE. Sleep modulation of epileptic activity in mesial and neocortical temporal lobe epilepsy: a study with depth and subdural electrodes. Epilepsy Behav. (2013) 28:185–90. 10.1016/j.yebeh.2013.04.010 23751358

[40] Watanabe Y Ogihara M Hoshika A. Cluster of epileptic spasms preceded by focal seizures observed in localization-related epilepsy. Brain Dev. (2007) 29:571–6. 10.1016/j.braindev.2007.03.006 17482399

[41] Malow BA Aldrich MS. Localizing value of rapid eye movement sleep in temporal lobe epilepsy. Sleep Medicine. (2000) 1:57–60. 10.1016/S1389-9457(99)00008-8 10733621

[42] Bazil CW Walczak TS. Effects of sleep and sleep stage on epileptic and nonepileptic seizures. Epilepsia. (1997) 38:56–62. 10.1111/j.1528-1157.1997.tb01077.x 9024184

[43] McKenzie MB Jones ML O'Carroll A Serletis D Shafer LA Ng MC. Breakthrough spikes in rapid eye movement sleep from the epilepsy monitoring unit are associated with peak seizure frequency. Sleep. (2019) 43:zsz281. 10.1093/sleep/zsz281 31768558

[44] Shouse MN Siegel JM Wu MF Szymusiak R Morrison AR. Mechanisms of seizure suppression during rapid-eye-movement (REM) sleep in cats. Brain Res. (1989) 505:271–82. 10.1016/0006-8993(89)91453-4 2598045 PMC9624451

[45] Frauscher B von Ellenrieder N Dubeau F Gotman J. EEG desynchronization during phasic REM sleep suppresses interictal epileptic activity in humans. Epilepsia. (2016) 57:879–88. 10.1111/epi.13389 27112123 PMC4949560

[46] Shouse MN Farber PR Staba RJ. Physiological basis: how NREM sleep components can promote and REM sleep components can suppress seizure discharge propagation. Clin Neurophysiol. (2000) 111(Suppl. 2):S9–18. 10.1016/S1388-2457(00)00397-7 10996550

[47] McCormick DA. Neurotransmitter actions in the thalamus and cerebral cortex and their role in neuromodulation of thalamocortical activity. Progr Neurobiol. (1992) 39: 337–88. 10.1016/0301-0082(92)90012-4 1354387

[48] Reinoso-Suárez F De Andrés I Rodrigo-Angulo ML Garzón M. Brain structures and mechanisms involved in the generation of REM sleep. Sleep Med Rev. (2001) 5:63–77. 10.1053/smrv.2000.0136 12531045

[49] España RA Scammell TE. Sleep neurobiology from a clinical perspective. Sleep. (2011) 34:845–58. 10.5665/SLEEP.1112 21731134 PMC3119826

[50] Ng MC. Orexin and epilepsy: potential role of REM sleep. Sleep. (2017) 40:zsw061. 10.1093/sleep/zsw061 28364414

[51] Steriade M Contreras D Amzica F. Synchronized sleep oscillations and their paroxysmal developments. Trends Neurosci. (1994) 17:201–7. 10.1016/0166-2236(94)90105-8 7520202

[52] Niedermeyer E. The Generalized Epilepsies: A Clinical Electroencephalographic Study. Springfield, IL: C. C. Thomas (1972). p. 247.

[53] Frauscher B von Ellenrieder N Ferrari-Marinho T Avoli M Dubeau F Gotman J. Facilitation of epileptic activity during sleep is mediated by high amplitude slow waves. Brain J Neurol. (2015) 138 (Pt 6):1629–41. 10.1093/brain/awv073 25792528 PMC4614129

[54] Haider B McCormick DA. Rapid neocortical dynamics: cellular and network mechanisms. Neuron. (2009) 62:171–89. 10.1016/j.neuron.2009.04.008 19409263 PMC3132648

[55] Crunelli V Hughes SW. The slow (1 Hz) rhythm of non-REM sleep: a dialogue between three cardinal oscillators. Nat Neurosci. (2010) 13:9–17. 10.1038/nn.2445 19966841 PMC2980822

[56] Warren CP Hu S Stead M Brinkmann BH Bower MR Worrell GA. Synchrony in normal and focal epileptic brain: the seizure onset zone is functionally disconnected. J Neurophysiol. (2010) 104:3530–9. 10.1152/jn.00368.2010 20926610 PMC3007634

[57] Pascual-Marqui RD Standardized low resolution brain electromagnetic tomography (sLORETA): technical details. Methods Findings Exp Clin Pharmacol. (2002) 24D:5–12. 12575463

[58] Wagner M Fuchs M Kastner J. Evaluation of sLORETA in the presence of noise and multiple sources. Brain Topogr. (2004) 16:277–80. 10.1023/B:BRAT.0000032865.58382.62 15379227

[59] Cosandier-Rimélé D Ramantani G Zentner J Schulze-Bonhage A Dümpelmann M. A realistic multimodal modeling approach for the evaluation of distributed source analysis: application to sLORETA. J Neural Eng. (2017) 14:056008. 10.1088/1741-2552/aa7db1 28677591

