# Supplementary material for: Can REM Sleep Localize the Epileptogenic Zone? A Systematic Review and Analysis
Source: Front Neurol. 2020 Jul 24;11:584. doi: 10.3389/fneur.2020.00584 (PMC7393443; doi:10.3389/fneur.2020.00584)
Supplement: Supplementary file 1 [file Data_Sheet_1.docx]

**Supplementary Information**

**Literature review**

We performed a broad literature search on REM sleep and epilepsy to seek original research characterizing spatial attributes or localizing value of epileptic phenomena in REM sleep. Search strategy developed by two authors (GAM, MCN) and was applied in PubMed, Scopus, and EMBASE. See Supplementary Figure 1 for exact search string used.

On March 12, 2020, 1,274 records were obtained from PubMed, 1,865 from EMBASE, and 2,279 from Scopus. Duplicates were identified and eliminated in [Mendeley Desktop 1.19.4](https://www.mendeley.com/?interaction_required=true), resulting in 3043 unique records remaining. As documented in manuscript Figure 3 according to Preferred Reporting Items for Systematic Reviews and Meta-Analyses (PRISMA), records were then screened for possible pertinence (GAM) according to following inclusion criteria:

1. Original research in human subjects.
2. Epileptic phenomena recorded in REM.
3. Spatial attributes of the epileptic phenomena in REM were analyzed.

Screening by these criteria caused 2893 records to be excluded while 150 remained. No full text or no English full text was available for another 35 records, causing their exclusion; meanwhile, 5 new records were uncovered by manual reference check of select pertinent articles. In total, full text assessment was performed on 120 records (GAM, MCN).

Of 120 records, we obtained 19 original research reports characterizing spatial attributes or localizing value of epileptic phenomena in REM sleep. These are catalogued in manuscript Table 1 and Figure 4.

Supplementary figure 1. Search strategy in PubMed.
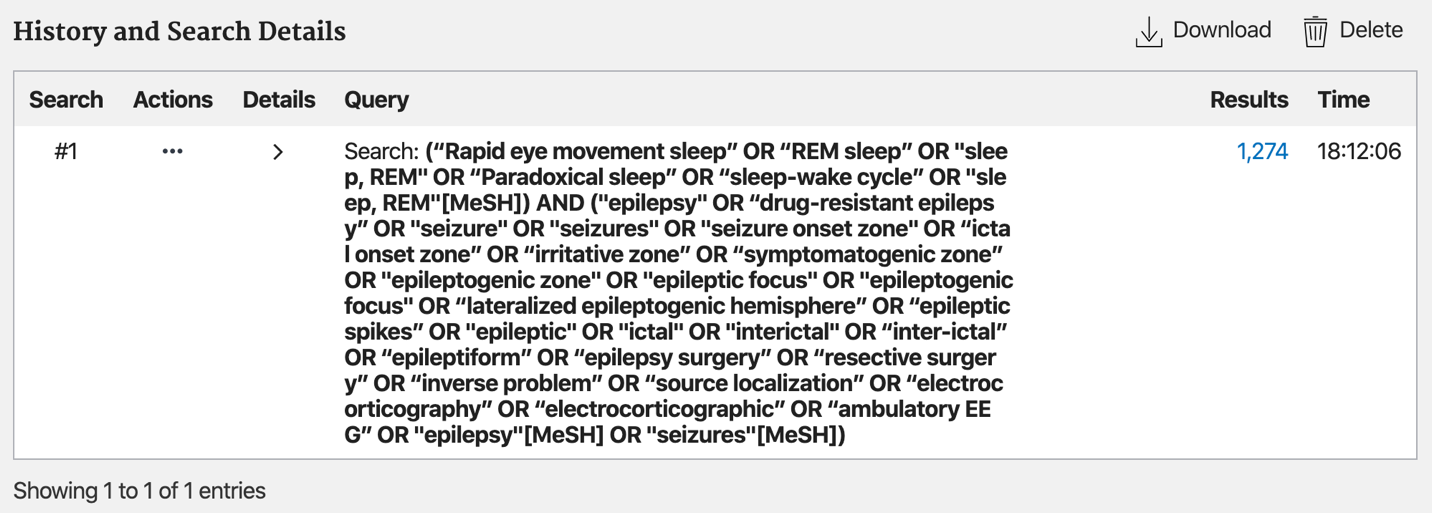


**Supplementary table S1. Interictal localization of REM in patients who continued to seize post-resective surgery (Engel outcomes II-IV).** Given ongoing post-operative seizures, localization of EZ is not validated. In these patients, concordance with EZ does not indicate correct localization.

| **Reference** | **Total Engel II-IV Patients (n)** | **REM concordant with EZ** | | **REM discordant with EZ** | | |
| --- | --- | --- | --- | --- | --- | --- |
|  |  | $\boldsymbol{\geq}$**1 other SWS also concordant**  **n (%)** | **0 other SWS concordant**  **n (%)** | **2 other SWS concordant**  **n (%)** | **1 other SWS concordant**  **n (%)** | **0 SWS concordant**  **n (%)** |
| **Sakuraba et al. 2016** | **6** | 1  (16.7) | 1  (16.7) | 0^a^ | 2  (33.3) | 2  (33.3) |
| **Ochi et al. 2011** | **5** | 3  (60) | 2^b^  (40)^b^ | 0 | 0 | 0 |
| **Montplaisir et al. 1987^b^** | **4** | 3  (75) | 0 | 0 | 1^c^  (25)^c^ | 0 |
| **Lieb et al. 1980** | **4** | 3  (75) | 0 | 0 | 0 | 1  (25) |
| **Percent average** (weighted per study) | | **56.7%** | **14.2%** | **0** | **14.6%** | **14.6%** |

^a^Wakefulness not analyzed. ^b^REM agree with side of ictal onset (n = 1) and largest tuber (n = 2). ^c^In NREM and wakefulness, ictal localization was considered.

**Supplementary table S2. REM’s effect on IED field size.**

| **Reference** | **Subjects with REM IEDs**  **(n)** | **Subjects with REM IEDs smaller spatial field**  **n (%)** |
| --- | --- | --- |
| **Kang et al. 2020** | **6** | 6 (100%) |
| **Ng 2017** | **39** | 6 (15.4%) |
| **Okanari et al. 2013** | **20** | 15 (75%) |
| **Percent average** (weighted by study) | | **63.5%** |
